# Binding of a C-type lectin’s coiled-coil domain to the Domeless receptor directly activates the JAK/STAT pathway in the shrimp immune response to bacterial infection

**DOI:** 10.1371/journal.ppat.1006626

**Published:** 2017-09-20

**Authors:** Jie-Jie Sun, Jiang-Feng Lan, Xiao-Fan Zhao, Gerardo R. Vasta, Jin-Xing Wang

**Affiliations:** 1 Shandong Provincial Key Laboratory of Animal Cells and Developmental Biology, School of Life Sciences, Shandong University, Jinan, Shandong, China; 2 Department of Microbiology and Immunology, School of Medicine, University of Maryland Baltimore and Institute of Marine and Environmental Technology, Baltimore, Maryland, United States of America; Stanford University, UNITED STATES

## Abstract

C-type lectins (CTLs) are characterized by the presence of a C-type carbohydrate recognition domain (CTLD) that by recognizing microbial glycans, is responsible for their roles as pattern recognition receptors in the immune response to bacterial infection. In addition to the CTLD, however, some CTLs display additional domains that can carry out effector functions, such as the collagenous domain of the mannose-binding lectin. While in vertebrates, the mechanisms involved in these effector functions have been characterized in considerable detail, in invertebrates they remain poorly understood. In this study, we identified in the kuruma shrimp (*Marsupenaeus japonicus*) a structurally novel CTL (MjCC-CL) that in addition to the canonical CTLD, contains a coiled-coil domain (CCD) responsible for the effector functions that are key to the shrimp’s antibacterial response mediated by antimicrobial peptides (AMPs). By the use of *in vitro* and *in vivo* experimental approaches we elucidated the mechanism by which the recognition of bacterial glycans by the CTLD of MjCC-CL leads to activation of the JAK/STAT pathway *via* interaction of the CCD with the surface receptor Domeless, and upregulation of AMP expression. Thus, our study of the shrimp MjCC-CL revealed a striking functional difference with vertebrates, in which the JAK/STAT pathway is indirectly activated by cell death and stress signals through cytokines or growth factors. Instead, by cross-linking microbial pathogens with the cell surface receptor Domeless, a lectin directly activates the JAK/STAT pathway, which plays a central role in the shrimp antibacterial immune responses by upregulating expression of selected AMPs.

## Author summary

The JAK/STAT pathway mediates the effects of a large number of cytokines and growth factors. It is activated following binding of a cytokine or growth factor to its respective receptor. To date, over 50 cytokines and growth factors have been shown to utilize the pathway to regulate cell growth, survival differentiation, motility, and immune responses. The JAK/STAT pathway is ubiquitous in vertebrates but can also be found as an intact pathway in some invertebrates, including shrimp. However, few cytokines and growth factors like molecules are identified in invertebrates and the function of the pathway in invertebrates is seldom studied. In this study, we identified core components of JAK/STAT pathway in shrimp and found the pathway had an important function in antibacterial immunity. Bacterial pathogens directly activate the JAK/STAT pathway through a secreted C-type lectin containing a coiled coil domain and a C-type lectin domain (MjCC-CL) in shrimp. Working as a cytokine like ligand, the MjCC-CL binds to polysaccharides from bacteria and the ILR domain of Domeless, induces STAT phosphorylation and translocation into the nucleus, and expression of several AMPs. The MjCC-CL, both as the pattern recognition receptor of bacteria and the ligand of Dome mediates activation JAK/STAT pathway.

## Introduction

Like other invertebrates, the shrimp’s defense against microbial pathogens relies on innate immune responses, which generally encompass their recognition, killing and disposal through humoral and cellular mechanisms. Humoral responses are rapid and effective, and include recognition factors such as lectins, as well as effector mechanisms, including hemolymph clotting, production of antimicrobial peptides (AMPs) and oxygen reactive intermediates, and, melanization of the microbe [1]. The cellular immune responses include pathogen recognition, killing and clearance by phagocytosis, immobilization by hemocyte extracellular traps, or nodulation or encapsulation of larger microorganisms [2, 3].

Initiation of the innate immune response is triggered by pathogen sensors called pattern recognition receptors (PRRs) such as Toll-like receptors and lectins. More than 10 different types of PRRs are found in shrimp [4]. Among these PRRs, C-type lectins (CTLs) are important in the recognition of carbohydrate moieties (microbe-associated molecular patterns, MAMPs) displayed on microbial surfaces. CTLs are a structurally diverse family of carbohydrate-binding proteins of wide taxonomic distribution in both vertebrates and invertebrates, characterized by calcium-dependent ligand recognition through a C-type carbohydrate recognition domain (CTLD) that displays a unique sequence motif and structural fold. By recognizing microbial glycans, the CTLD is responsible for the CTLs’ roles as pattern recognition receptors (PRRs) in the innate immune response to bacterial infection of both vertebrates and invertebrates. In addition to the CTLD, however, most CTLs display additional domains that can carry out effector functions. In CTLs from vertebrates some of these effector functions have been well established and the mechanisms involved have been described in detail. One of the most thoroughly characterized example, the mannose-binding lectin (MBL) a prototypical CTL, displays a collagenous domain that by associating with an MBL-associated serine protease (MASP) can activate the complement cascade [5, 6]. In contrast, the effector functions of CTLs from invertebrates remain poorly understood. During the past few years, we have focused on the roles of CTLs in the shrimp immune response to bacterial infection [7–9]. Based on their domain organization, at present time the shrimp CTLs can be classified in three distinct groups: (1) CTLs that contain only one CTLD, (2) CTLs with two CTLDs, and (3) CTLs with one CTLD and an additional domain [7] such as a low-density lipoprotein receptor class A domain [10], and an immunoglobulin-like domain [11]. The shrimp CTLDs can recognize viral or bacterial glycans, and thus immobilize the invading microorganisms, CTLs can also induce downstream immune responses aimed at their killing, destruction, and elimination. For some shrimp CTLs, their effector innate immune functions such as phagocytosis, prophenoloxidase activation, and promotion of respiratory burst have been described [7, 8], although the mechanisms involved still remain unclear. We recently identified in the kuruma shrimp *Marsupenaeus japonicus* a novel CTLs (MjHeCL) that in addition to recognizing microbial glycans via the CTLD, displays unique functional properties, as the inhibition of proliferation of the hemolymph microbiota by maintaining the homeostatic expression of antimicrobial peptides [7]. The mechanistic aspects of this regulation of AMP expression in shrimp by CTLs, however, remained to be elucidated.

In this study, we identified in *M*. *japonicus* a novel CTL that we designated MjCC-CL, and that like MjHeCL, can regulate the expression of selected AMPs. MjCC-CL has a chimeric structure comprising the canonical CTLD that can recognize bacterial glycans and a coiled-coil domain (CCD). Our study revealed that the mechanism by which upon bacterial challenge, MjCC-CL regulates AMP expression is based on signaling by the JAK/STAT (Janus Kinase/Signal Transducer and Activator of Transcription) that leads to the transcription of five AMPs. The JAK/STAT pathway, which is common to both invertebrates and vertebrates, controls multiple biological processes, including cell development, growth and survival, tissue homeostasis and immune responses [12, 13]. In vertebrates, the JAK/STAT pathway is indirectly activated by cytokines or growth factors released upon stress signals and cell death. In striking contrast, the present study revealed that activation of the JAK/STAT pathway and AMP upregulation in shrimp takes place by direct MjCC-CL-mediated recognition of the microbial pathogen glycans by the CTLD, and binding of the CCD to the hemocyte surface Domeless, the receptor that activates JAK/STAT pathway.

## Results

### The shrimp C-type lectin MjCC-CL participates in antibacterial immunity

**Fig 1:**
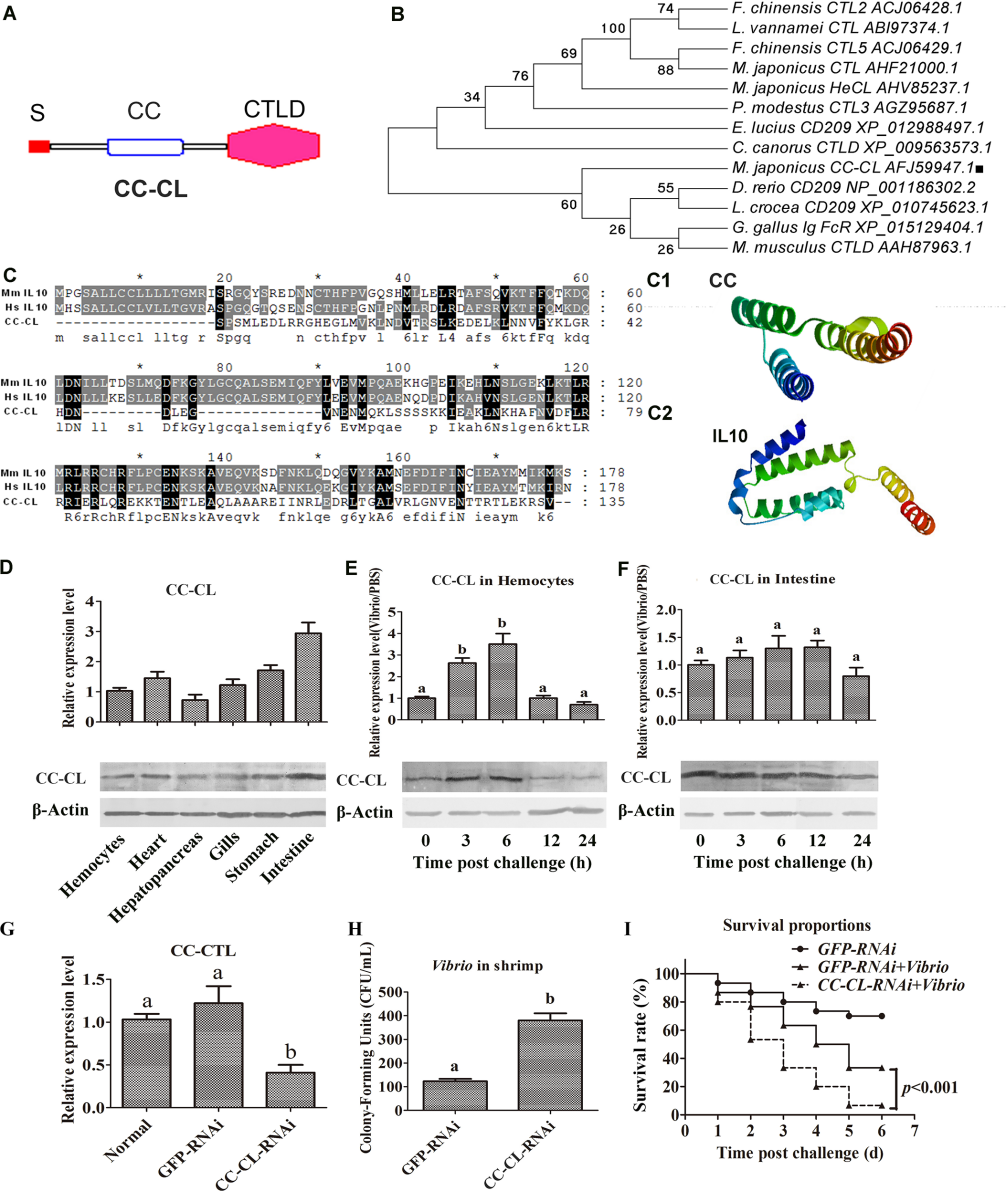
The shrimp C-type lectin MjCC-CL participates in antibacterial immunity. (**A**) The domain atchitecture of shrimp MjCC-CL predicted by SMART (http://www.smart.embl-heidelberg.de/). (**B**) Phylogenetic analysis of MjCC-CL with other CTLs from various species. All amino acid sequences were collected from NCBI, and the tree was constructed with MEGA 6 software. MjCC-CL is shown with a black square frame in the tree. (**C**) Multiple alignments of the N-terminal CC region of MjCC-CL with Hs IL10 (*Homo sapiens* interleukin 10, CAG46790.1) and Ms IL10 (*Mus musculus* interleukin 10, EDL39722.1). (**C1**) The structure of the N-terminal CC region of MjCC-CL was analyzed using the online SWISS-MODEL server. (**C2**) The structure of human IL10 was analyzed using the online SWISS-MODEL server. (**D**) The tissue distribution of MjCC-CL was analyzed by qRT-PCR and western blotting. β-actin was used as a control. (**E-F**) qRT-PCR and western blotting were used to detect the time course of MjCC-CL expression in hemocytes (E) and intestine (F) after challenge with *V*. *anguillarum*. (**G**) RNAi efficiency after *dsMjCC-CL* injection was determined using qRT-PCR. (**H**) Bacterial scavenging capacity of shrimp after RNAi against MjCC-CL. (**I**) The survival rate of *MjCC-CL*-RNAi shrimp after infection with *V*. *anguillarum*. *GFP*-RNAi was used as a control. Differences among the groups were analyzed using a *t*-test (*p < 0*.*05*) or with one-way ANOVA analysis; asterisks indicate significant differences (*p < 0.*05*, **p < 0.*01*).

From our transcriptome analysis of the kuruma shrimp (*M*. *japonicus*) we identified a unique CTL which we named MjCC-CL (GenBank accession no. KU213612), and comprises a signal peptide, a coiled-coil domain (CCD), and a CTLD (Fig 1A). A phylogenetic analysis of MjCC-CL and other CTLs from invertebrate and vertebrate species showed that in contrast with MjHeCL, a shrimp CTL that we recently characterized [7], MjCC-CL clusters with CTLs from vertebrates (Fig 1B). Further SMART analysis indicated that the N-terminal region (including the CCD) of MjCC-CL is similar to the interleukin (IL) 10 domain from mammals at the threshold level. Multiple alignment of the N-terminal region of MjCC-CL with Hs-IL10 (*Homo sapiens* interleukin 10, CAG46790.1) and Ms-IL10 (*Mus musculus* interleukin 10, EDL39722.1) showed that they share low similarity (Fig 1C). A comparison of homology models of the CCD of MjCC-CL and IL10 revealed an α-helix-rich structure for the CCD (Fig 1C1), with overall similarity to IL10 (Fig 1C2). The tissue distribution of MjCC-CL transcripts and protein in the control and challenged animals were analyzed by qRT-PCR and western blotting, respectively. The results in control shrimp showed that MjCC-CL was expressed in all tissues tested, with a relatively high expression in the intestine (Fig 1D). In the challenged shrimp, a time-course expression analysis showed that MjCC-CL was increased significantly in hemocytes at 3 and 6 h after *V*. *anguillarum* injection (Fig 1E), and with no obvious changes in the relatively high level of expression in intestine (Fig 1F). To analyze *in vivo* the potential immune function of MjCC-CL, we knocked down *MjCC-CL* expression by RNAi (*MjCC-CL*-RNAi shrimp) (Fig 1G) and assessed the bacterial clearance and survival rate of shrimp relative to the control animals with normal *MjCC-CL* expression. The results showed that in the experimentally challenged *MjCC-CL*-RNAi shrimp the number of bacteria increased significantly (Fig 1H), while the survival rate of the shrimp decreased significantly (Fig 1I) as compared to the controls. These results strongly suggested that MjCC-CL participates in the antibacterial response in shrimp.

### MjCC-CL binds to bacterial surface components and to the shrimp hemocyte surface

**Fig 2:**
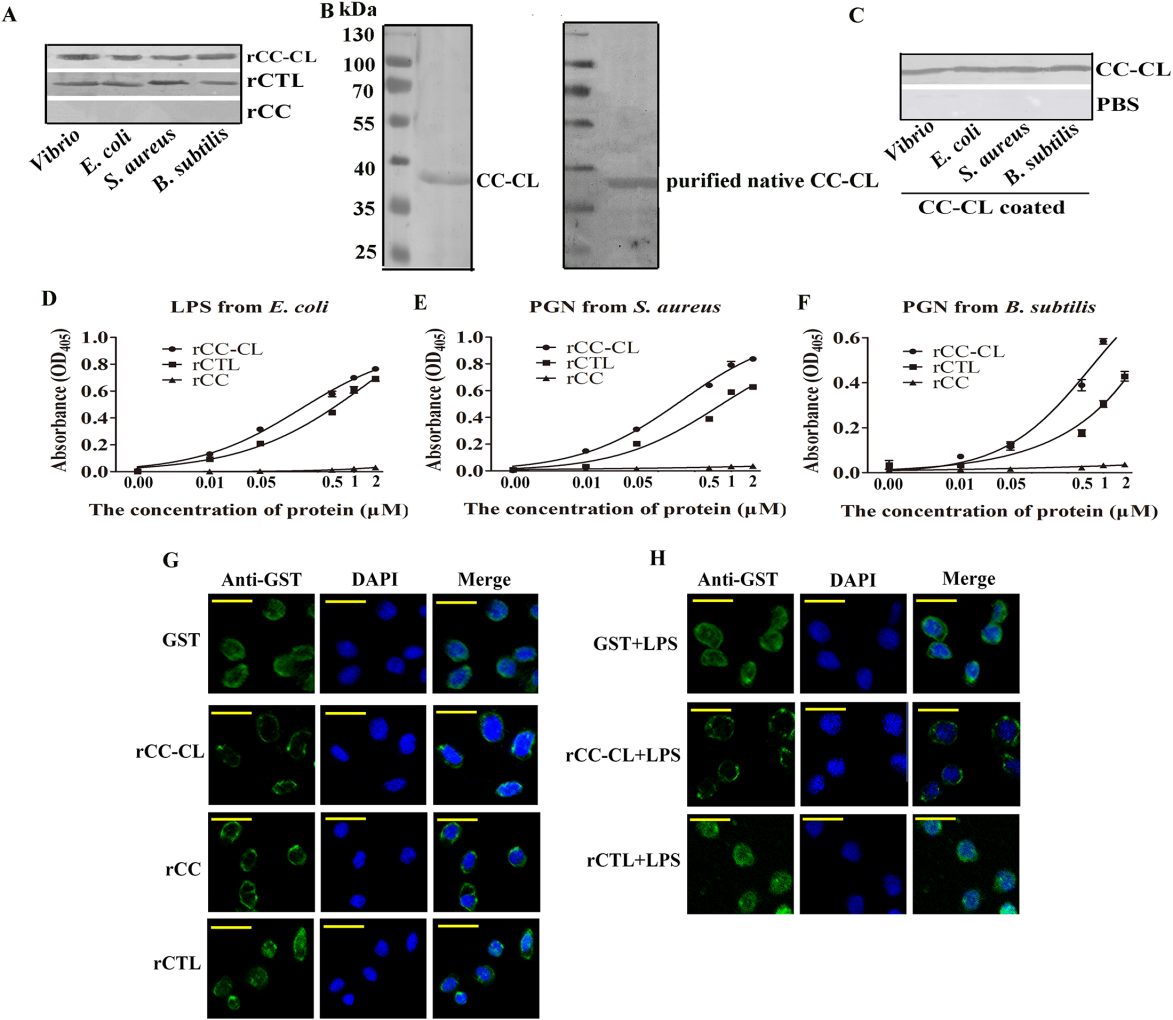
MjCC-CL binds to bacteria and to the hemocyte surface. (**A**) Western blotting was used to analyze the binding activity of rMjCC-CL, the rCTLD of MjCC-CL and the rCCD of MjCC-CL with different bacteria. (**B**) The left panel showed the molecular masses of native MjCC-L as detected with MjCC-CL polyclonal antibody by western blot. In the right panel, Native MjCC-CL was purified from kuruma shrimp intestine with CNBr-activated Sepharose 4B by coupling the antibody from MjCC-CL. (**C**) Western blotting was used to analyze the binding activity of native MjCC-CL with different bacteria. (**D-F**) An ELISA was performed to detect the binding activity of rMjCC-CL, the rCTLD and the rCCD to LPS from *E*. *coli* and PGN from *S*. *aureus* or *B*. *subtilis*. (**G**) Immunocytochemical analysis was performed to test the binding of rMjCC-CL and rCC to the hemocytes surface. (H) Immunocytochemical assay was performed to detect if rMjCC-CL can cross-link LPS to the hemocyte surface.

To gain further insight into the role(s) of MjCC-CT in antibacterial immunity, we analyzed the potential binding to both Gram-positive (GP) and Gram-negative (GN) bacteria of: (a) the intact recombinant MjCC-CL (S1B Fig); (b) the separate recombinant CC and CTL domains (S1C and S1D Fig); and (c) the authentic MjCC-CL purified from shrimp intestine. The results showed that the intact rMjCC-CL and the rCTLD bound to the GP bacteria *S*. *aureus* and *B*. *subtilis*, and to the GN bacteria *V*. *anguillarum* and *E*. *coli*, whereas the rCCD showed no binding activity to any of the bacterial species tested (Fig 2A). Like the rMjCC-CL, the native MjCC-CL purified from shrimp intestine (Fig 2B) also bound to four types of bacteria (Fig 2C). Direct binding analysis with enzyme-linked immunosorbent assay (ELISA) revealed that the rMjCC-CL and CTLD bound to lipopolysaccharide (LPS) from *E*. *coli* and peptidoglycan (PGN) from *S*. *aureus* or *B*. *subtilis*, whereas no binding activity was detected for the rCCD (Fig 2D–2F and S1 Table). We then examined the potential binding of MjCC-CL to the shrimp hemocyte surface by immunocytochemical analysis using an anti-GST antibody. The results showed that rMjCC-CL or rCC could bind to the hemocyte surface (Fig 2G). To detect if the LPS/rMjCC-CL complexes could bind to the hemocyte surface, rMjCC-CL that had been pre-incubated with LPS was injected into shrimp, and the hemocytes were collected for immunocytochemical analysis. The control animals received LPS-incubated GST instead of LPS/rMjCC-CL complexes. The results revealed that the injected LPS/rMjCC-CL complexes localized on the hemocyte external surface, whereas in the GST control animals the label localized to the hemocyte cytoplasm (Fig 2G and 2H). Taken together, the results indicate that MjCC-CL not only binds to both GN and GP bacteria by recognizing their cell surface polysaccharides, but also the “self” ligands on the shrimp hemocyte surface.

### MjCC-CL regulates the expression of AMPs

**Fig 3:**
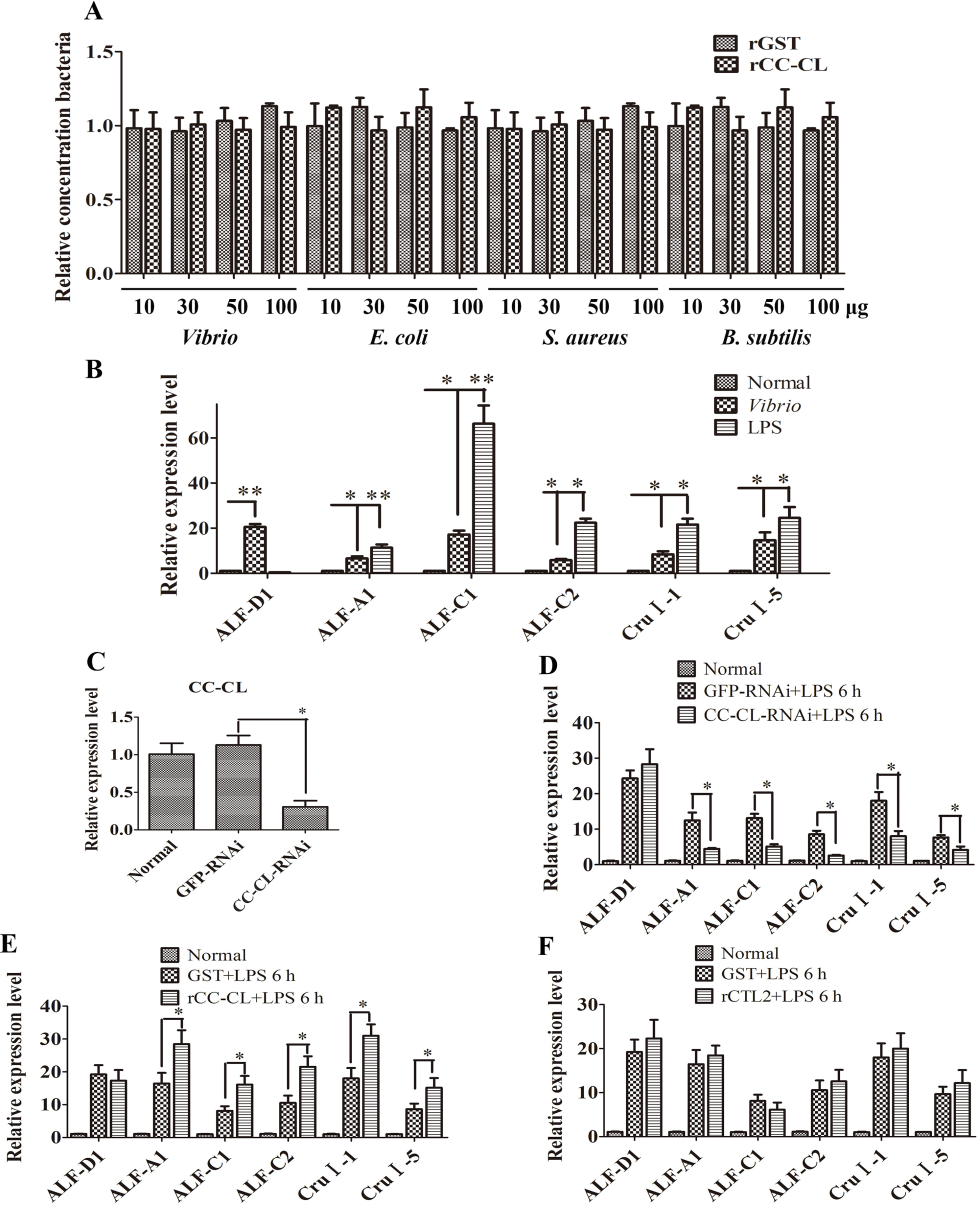
MjCC-CL regulates the expression of AMPs. (A) The rMjCC-CL has no direct bacteriostatic/bacteriocidal activity *in vitro*. The rMjCC-CL was incubated with *V*. *anguillarum*, *E*. *coli*, *S*. *aureus* or *B*. *subtilis* for 24 h. Bacterial growth was evaluated by measuring the absorbance at 600 nm. GST protein was used as control. (**B**) The expression patterns of 6 AMPs (*ALF-A1*, *ALF-C1*, *ALF-C2*, *ALF-D1*, *CruI-1* and *CruI-5*) in the intestine of shrimp challenged with *V*. *anguillarum* or LPS were analyzed by qRT-PCR. (**C**) RNAi efficiency after *dsMjCC-CL* injection was determined using qRT-PCR. (**D**) The expression of *ALF-A1*, *ALF-C1*, *ALF-C2*, *ALF-D1*, *CruI-1* and *CruI-5* in *MjCC-CL*-RNAi shrimp challenged by LPS was detected with qRT-PCR. *GFP*-RNAi was used as control. (**E**) The expression of *ALF-A1*, *ALF-C1*, *ALF-C2*, *ALF-D1*, *CruI-1* and *CruI-5* in rMjCC-CL-injected shrimp was analyzed by qRT-PCR. GST was used as a control. (**F**) The expression of *ALF-A1*, *ALF-C1*, *ALF-C2*, *ALF-D1*, *CruI-1* and *CruI-5* in rCTL2-injected shrimp was analyzed by qRT-PCR. GST was used as control. Differences among the groups were analyzed using a *t*-test (*p < 0.05, **p < 0.01).

As MjCC-CL binds to the bacterial surface, we next examined the possibility that this shrimp lectin could have direct bacteriostatic or bacteriocidal activity. Exposure of bacterial cultures to intact rMjCC-CL at increasing concentrations ranging from 10 μg/ml to 100 μg/ml showed no differences in bacterial growth with control cultures that received no MjCC-CL (Fig 3A). In light of these results we investigated if the antibacterial activity of MjCC-CL could be indirect, such as *via* the upregulation of AMP expression. For this, we first evaluated the expression of six AMPs, including 4 antilipopolysaccharide factors (ALFs) (GenBank accession nos. ALF-A1 KU213607, ALF-C1 KU213608, ALF-C2 KU160498, and ALF-D1 KU160499) and 2 Crustins (Crus) (CruΙ-1 KU160502, and CruΙ-5 KU213606), in shrimp intestine after *V*. *anguillarum* or LPS challenge; the results showed that the expression of all six AMPs was significantly upregulated (Fig 3B). Then, we performed RNA interference (RNAi) of MjCC-CL and the expression of these six AMPs upon LPS challenge was analyzed. After transfection with RNAi targeting *MjCC-CL* in shrimp (Fig 3C) following challenge with LPS, no upregulation of expression of *ALF-A1*, *ALF-C1*, *ALF-C2*, *CruΙ-1* and *CruΙ-5* was observed. In contrast, expression of *ALF-D1* was equally upregulated in the RNAi transfected animals and in the control animals in which *MjCC-CL* was normally expressed (Fig 3D). To confirm the above results, the RNAi-transfected shrimp were first injected with rMjCC-CL protein, and subsequently challenged with LPS as above, and the expression of six AMPs was analyzed. The results showed that *ALF-A1*, *ALF-C1*, *ALF-C2*, *CruΙ-1* and *CruΙ-5* expression was increased significantly by LPS challenge in the rMjCC-CL-rescued shrimp, as compared to the control animals (Fig 3E). To confirm the specificity of the rMjCC-CL-mediated rescue we also injected a group of RNAi-transfected shrimp with recombinant CTL2, another shrimp CTL, and carried out the LPS challenge as above. The results showed that AMP expression was not induced significantly after rCTL2 injection, as compared to the control (Fig 3F). All the above results indicated that MjCC-CL specifically upregulates the expression of five different AMPs.

### MjCC-CL induces STAT translocation into nucleus

**Fig 4:**
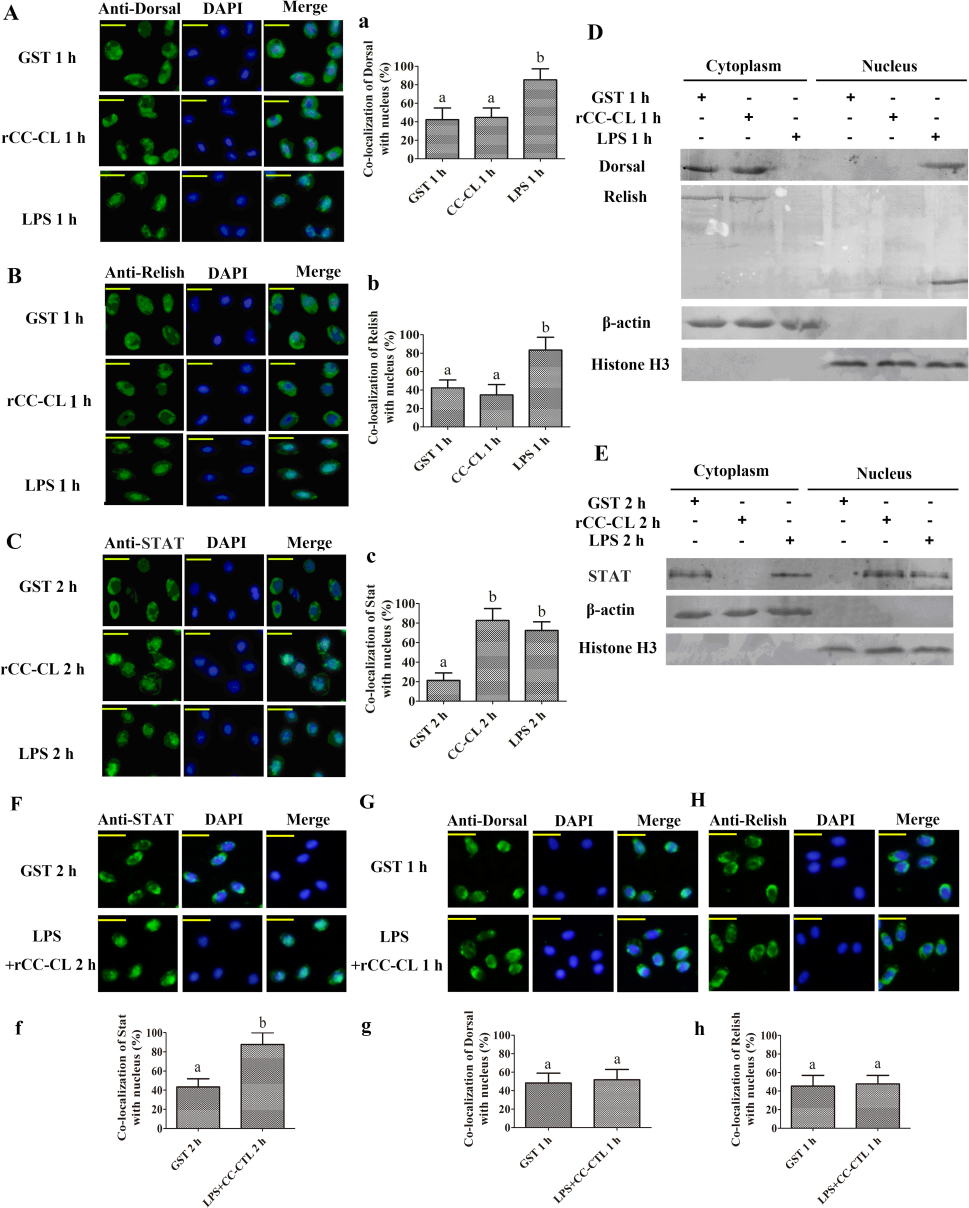
MjCC-CL induces STAT (but not Dorsal or Relish) translocation into nucleus. (**A**). Dorsal translocation into the nucleus in hemocytes was detected in rMjCC-CL-injection shrimp. GST and LPS were separately used as negative and positive control. (**a**) Statistic analysis of co-localization of Dorsal with nucleus with WCIF ImageJ software. (**B**) Relish translocation into the nucleus in hemocytes was detected in rMjCC-CL-injection shrimp. GST and LPS were separately used as negative and positive control. (**b**) Statistical analysis of co-localization of Relish with nucleus. (**C**) STAT translocation into the nucleus in hemocytes was detected in rMjCC-CL-injection shrimp. (**c**) Statistical analysis of C. (**D**) The subcellular distribution of Dorsal and Relish in intestine was detected by western blotting after rMjCC-CL-injection. (**E**) The subcellular distribution of STAT in intestine was detected by western blotting after rMjCC-CL-injection. β-actin and histone H3 were used as loading controls for the cytoplasmic or nuclear proteins. GST and LPS were separately used as negative and positive control. (**F-H**) Recombinant MjCC-CL bound with LPS was injected into shrimp, STAT (F) Dorsal (G), and Relish (H) translocation into the nucleus in hemocytes were detected by immunocytochemical assay. GST was used as negative control. (**f-h**) Statistic analyses of co-localization of STAT, Dorsal and Relish with nucleus using WCIF ImageJ software. Different letters indicate significant differences (*p* < 0.05) in one-way ANOVA analysis.

**Fig 5:**
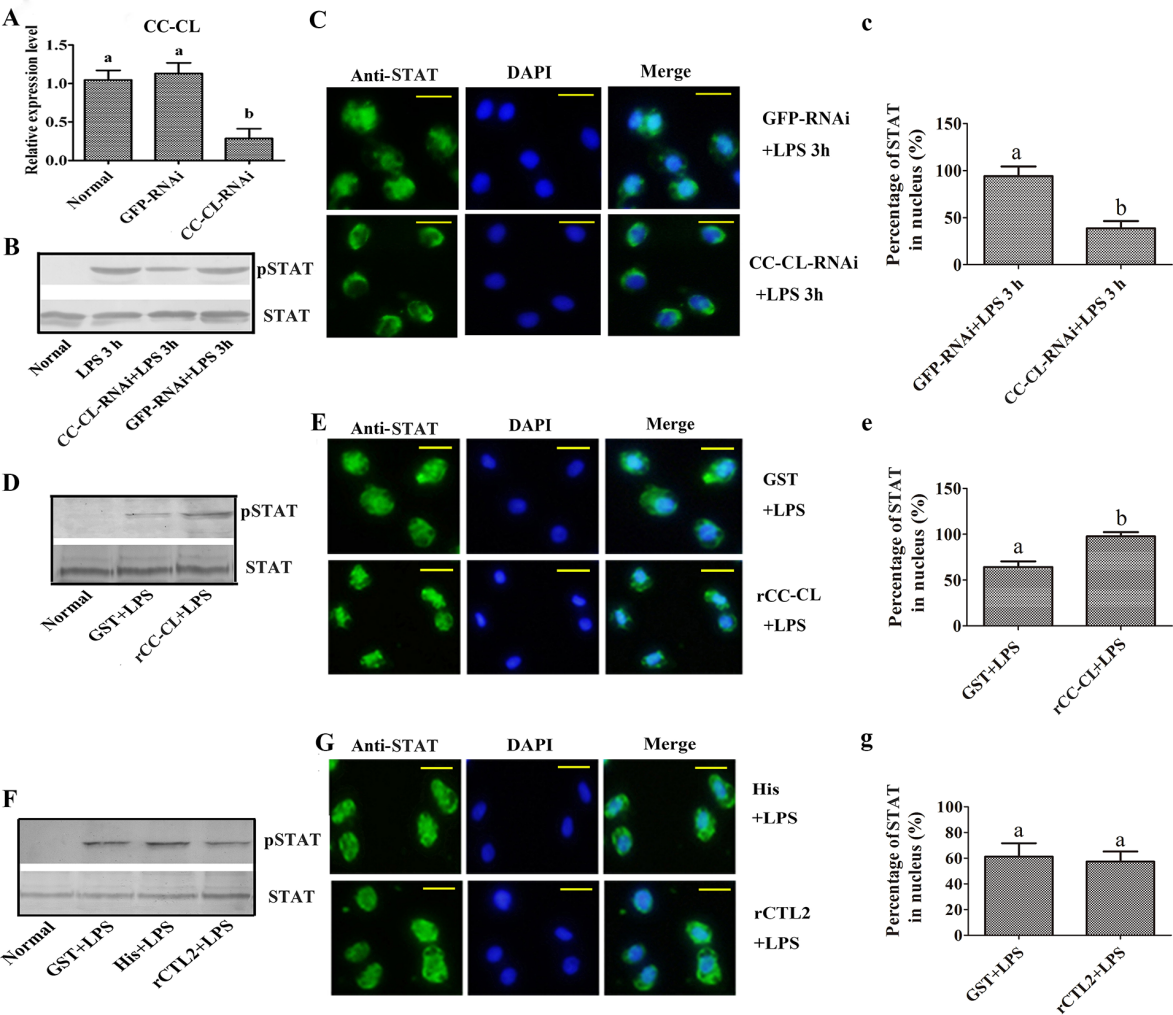
MjCC-CL mediates activation of JAK/STAT signaling. (**A**) The RNAi efficiency of *MjCC-CL* was analyzed by qRT-PCR. (**B**) STAT phosphorylation in *MjCC-CL*-silenced-shrimp was detected by western blotting. (**C**) STAT translocation in the hemocytes of *MjCC-CL*-silenced shrimp was detected with an immunocytochemical assay with a STAT-specific antibody. (**c**) Statistical analysis of C: the percentage of cells with translocated STAT in the nucleus was analyzed using WCIF ImageJ software. (**D**) STAT phosphorylation in rMjCC-CL-injected shrimp was determined by western blotting. (**E**) STAT translocation in the hemocytes of rMjCC-CL-injected shrimp was detected using an immunocytochemical assay with a STAT-specific antibody. (**e**) Statistical analysis of E: the localization of STAT within the nucleus was analyzed using WCIF ImageJ software. (**F**) STAT phosphorylation in rCTL2-injected shrimp was determined by western blotting (CTL2 is a C-type lectin from *M*. *japonicus* without a CCD that was used as a control). (**G**) STAT translocation in the hemocytes of rCTL2-injected shrimp was detected with an immunocytochemical assay with a STAT-specific antibody. (**g**) Statistical analysis of G: the localization of STAT within the nucleus of hemocytes was analyzed using WCIF ImageJ software. Different letters indicate significant differences (*p* < 0.05) in one-way ANOVA analysis.

Next, we investigated the signaling pathway through which MjCC-CL regulates AMP expression. In *Drosophila*, AMP expression is mainly regulated by the Toll and IMD pathways [14]. Additionally, the cytokine-activated JAK/STAT pathway is key for antiviral responses in both *Drosophila* and mammals [13, 15–18]. Since in mammals the JAK/STAT signaling pathway is activated by different cytokines, our observation that MjCC-CL contains an IL10-like domain led us to hypothesize that its function might be also related to this signaling pathway. Therefore, we examined the potential role(s) of MjCC-CL in activation of the three signaling pathways, Toll, IMD, and JAK/STAT by assessing the translocation into the hemocyte nucleus of their transcription factors Dorsal, Relish and STAT (GenBank accession no. KU213611), respectively, upon increasing circulating MjCC-CL levels, and using LPS challenge as a positive control. The results of immunocytochemical analysis of hemocytes from experimental and control shrimp showed that while LPS challenge induced Dorsal or Relish translocation (Fig 4A and 4B) no translocation of Dorsal (Fig 4A, a) or Relish (Fig 4B, b) was detected in rMjCC-CL-injected shrimp. In contrast, STAT did translocate into the hemocyte nucleus in both rMjCC-CL-injected and LPS-injected shrimp (Fig 4C, c). Western blot analysis of proteins extracted from cytoplasm and nucleus of intestinal cells yielded results similar to those from the immunocytochemical study (Fig 4D and 4E). To further confirm the results, LPS was first incubated with rMjCC-CL and upon which any remaining free LPS was washed off. The LPS-rMjCC-CL mixture was injected into shrimp and translocation of Dorsal, Relish and STAT into the hemocyte nucleus was examined. The results showed that the LPS-rMjCC-CL complex could induce STAT translocation into the hemocyte nucleus (Fig 4F-f) but not translocation of Dorsal (Fig 4G) or Relish (Fig 4H-h). To confirm the role of MjCC-CL in activation of the JAK/STAT pathway, we analyzed STAT phosphorylation in *MjCC-CL*-RNAi shrimp. The results showed that in the *MjCC-CL* knockdown shrimp (Fig 5A) the LPS challenge reduced STAT phosphorylation (Fig 5B) and inhibited STAT translocation into the nucleus (Fig 5C and c). When rMjCC-CL protein was injected into the *MjCC-CL* knockdown shrimp, the LPS challenge induced STAT phosphorylation (Fig 5D) and translocation into the nucleus (Fig 5E and e) as in the control shrimp. However, if in the rescue experiments rMjCC-CL was replaced by rCTL2, STAT phosphorylation (Fig 5F) and translocation did not markedly change (Fig 5G, g). Taken together, our results suggest that MjCC-CL regulates AMP expression via the JAK/STAT pathway, without involving the Toll or IMD pathways.

### The JAK/STAT pathway regulates AMP expression

**Fig 6:**
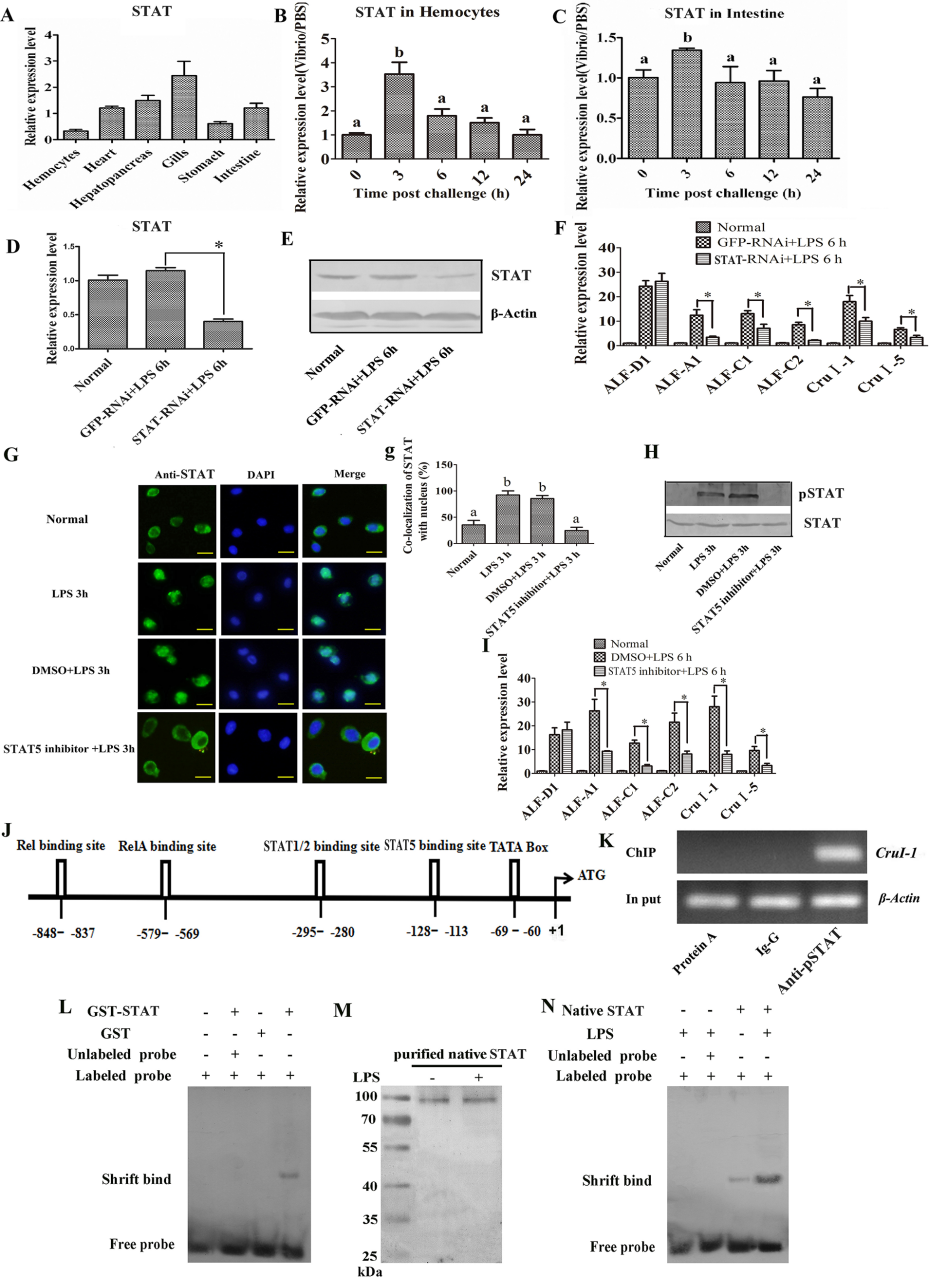
JAK/STAT pathway regulates expression of the same AMPs modulated by MjCC-CL. (A) The tissue distribution of STAT was analyzed with qRT-PCR. (B-C) The expression patterns of STAT in hemocytes (B) and intestine (C) were analyzed by qRT-PCR. (D) RNAi efficiency after *dsSTAT* injection was determined using qRT-PCR. (E) RNAi efficiency after *dsSTAT* injection was determined by western blotting. (F) The expression of *ALF-A1*, *ALF-C1*, *ALF-C2*, *ALF-D1*, *CruI-1* and *CruI-5* in *STAT*-RNAi shrimp challenged with LPS was determined by qRT-PCR. *GFP*-RNAi was used as a control. (G, g) STAT translocation into the nucleus in hemocytes was inhibited by the injection of a STAT5 inhibitor. (g) Statistical analysis of G: the localization of STAT in the nucleus of hemocytes was analyzed with WCIF ImageJ software. (H) STAT phosphorylation in the intestine was determined by western blotting after injection with a STAT5 inhibitor. (I) The expression of *ALF-A1*, *ALF-C1*, *ALF-C2*, *ALF-D1*, *CruI-1* and *CruI-5* in the intestine of shrimp injected with a STAT5 inhibitor was analyzed by qRT-PCR. (J) Genome walking was used to obtain the genomic sequence of *CruΙ-1*. The *CruΙ-1* genomic sequence contains NF-κB (Rel/RelA) and STAT family protein binding sites, a TATA box and a transcriptional start site. (K) ChIP and RT-PCR were used to detect STAT binding to the *CruΙ-1* promoter sequence. (L) EMSA detected a normal shifted band corresponding to STAT bound to the Dig-labeled CruΙ-1 probe. GST protein was used as a control. An unlabeled sequence of the CruΙ-1 probe was used as a competitor. (M) Native STAT was purified from intestine of untreated or LPS challenged kuruma shrimp using CNBr-activated Sepharose 4B by coupling the STAT antibody. (N) EMSA using native STAT protein purified from the intestine of shrimp challenged with LPS using CNBr-activated Sepharose 4B resin. Differences among the groups were analyzed using a *t*-test (*p* < 0.05) or with one-way ANOVA analysis; asterisks indicate significant differences (*p < 0.05, **p < 0.01).

We subsequently investigated whether the JAK/STAT pathway regulates AMP expression in shrimp. The shrimp STAT contains an N-terminal domain (NTD), a coiled-coil domain (CC), a DNA-binding domain (DB), a linker domain (LD), an SH2 domain (SH2) and a transactivation domain (TAD) (S2A Fig), and in a phylogenetic analysis, it clusters with other invertebrate STATs (S2B Fig). Analysis of the spatiotemporal distribution of shrimp STAT revealed that it was expressed in all tested tissues, but had relatively low expression in the hemocytes and stomach (Fig 6A). Expression of *STAT* was upregulated in hemocytes and the intestine at 3 h after bacterial challenge and gradually returned to normal levels from 6 to 24 h (Fig 6B and 6C). To investigate whether expression of *ALF-A1*, *ALF-C1*, *ALF-C2*, *CruΙ-1* and *CruΙ-5* is regulated by the JAK/STAT pathway, we knocked down *STAT* expression by RNAi (Fig 6D and 6E), and analyzed AMP expression. The results showed that the expression of the above five AMPs was reduced significantly in the intestine of *STAT*-RNAi shrimp (Fig 2F), a finding that is similar to the effect observed in *MjCC-CL*-RNAi shrimp. To confirm that the JAK/STAT pathway regulates AMP expression, we injected the shrimp with a STAT inhibitor prior to the LPS challenge, and analyzed STAT translocation and AMP expression. The results showed that in the STAT inhibitor-injected shrimp STAT did not translocate into the nucleus of the hemocytes upon LPS challenge (Fig 6G and g), and STAT phosphorylation in the intestine was also inhibited (Fig 6H). Further, in the STAT inhibitor-injected shrimp the LPS challenge failed to upregulate AMP expression (Fig 2I). These results suggest that STAT phosphorylation and translocation are functionally related to the increased expression of *ALF-A1*, *ALF-C1*, *ALF-C2*, *CruΙ-1 and CruΙ-5*.

To identify putative STAT-binding sites in the promoter sequences of the AMPs of interest we used a genome walking approach. We succeeded in identifying putative NF-κB and STAT-binding sites (Fig 6J) in the CruΙ-1 promoter sequence (S3 Fig). Next, we conducted a chromatin immunoprecipitation (ChIP) assay with anti-pSTAT, purified and analyzed the DNA fragment obtained, and amplified by RT-PCR the *CruI-1* sequence of interest (Fig 6K). Subsequently we carried out an electrophoretic mobility shift assay (EMSA) with a Dig-labeled CruΙ-1 probe containing the predicted STAT binding site, and purified the recombinant GST-STAT protein and native STAT protein from LPS-injected shrimp to confirm whether STAT directly binds to the predicted STAT binding site in the promoter sequence of *CruΙ-1*. These results showed that STAT could bind to the predicted STAT binding site in the *CruΙ-1* promoter sequence (Fig 6L and 6N) and that the binding ability was increased in LPS-injected shrimp (Fig 6N). All these results suggested that the JAK/STAT pathway regulates the expression of AMPs.

### The JAK/STAT pathway plays an important role in antibacterial immunity in shrimp

**Fig 7:**
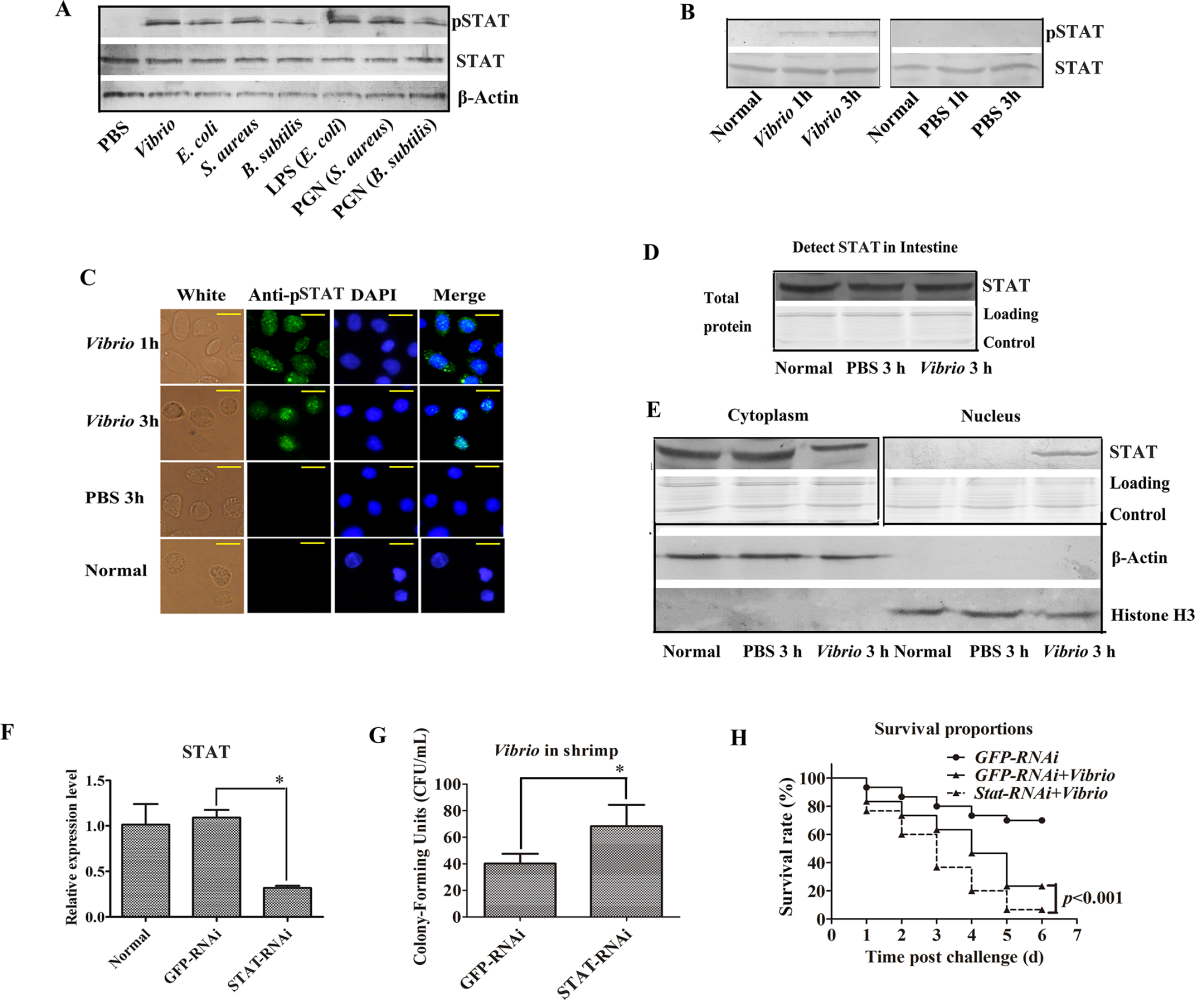
The JAK/STAT pathway plays an important role in shrimp antibacterial immunity. (**A**) Seven groups of shrimp were challenged with *V*. *anguillarum*, *E*. *coli*, *S*. *aureus*, *B*. *subtilis*, LPS from *E*. *coli*, PGN from *S*. *aureus* or *B*. *subtilis* and PBS, intestine proteins were extracted at 3 h, and STAT phosphorylation was analyzed by western blotting using a pSTAT antibody; total STAT and β-actin were detected with the corresponding antibodies (STAT or β-actin antibody) and were used as controls. (**B**) Detection of STAT phosphorylation in the intestine of shrimp 1 and 3 h after *V*. *anguillarum* challenge. PBS injection was used as a control. (**C**) STAT translocation in hemocytes was detected using an immunocytochemical assay with the pSTAT antibody. The PBS-injected shrimp and untreated shrimp were used as controls. (**D**) Total protein or the cytoplasmic or nuclear proteins were extracted from the intestine of shrimp challenged with *V*. *anguillarum* and used for western blot analysis with a STAT antibody. (**E**) The subcellular distribution of STAT in the intestine was detected by western blotting. β-actin antibody and histone H3 antibody were used as loading controls for the cytoplasmic or nuclear proteins. PBS injection was used as the control. (**F**) The efficiency of *STAT-*RNAi was analyzed by qRT-PCR. (**G**) The bacterial scavenging capacity of the shrimp after *STAT-*RNAi. *dsGFP* injection was used as the control. (**H**) The survival rate of *STAT*-RNAi shrimp infected with *V*. *anguillarum*. *GFP*-RNAi was used as the control. Differences among the groups were analyzed using a *t*-test (*p < 0.05).

To investigate the antibacterial function of the JAK/STAT pathway in shrimp, we first evaluated signaling activation by detecting STAT phosphorylation with an antibody specific for phosphorylated STAT (anti-pSTAT) after challenge with bacteria (*V*. *anguillarum*, *Escherichia coli*, *Staphylococcus aureus* or *Bacillus subtilis*), LPS (*E*. *coli*), or PGN (*S*. *aureus* or *B*. *subtilis*). The results showed that they all could induce STAT phosphorylation in the intestine 3 h post challenge, whereas no pSTAT could be detected in the intestine of control shrimp challenged with PBS (Fig 7A). As both GN and GP bacteria induced STAT phosphorylation, only the shrimp pathogen *V*. *anguillarum* and purified LPS were used in subsequent experiments. To expand the above results, we conducted a time-course analysis by assessing STAT phosphorylation in the intestine of shrimp at 1h and 3 h upon *V*. *anguillarum* challenge. The results revealed that the STAT phosphorylation increased in the intestine of *V*. *anguillarum-*challenged shrimp from 1 to 3 h after challenge (Fig 7B). We also examined STAT phosphorylation and translocation in hemocytes by immunocytochemical analysis, and the results indicated that pSTAT translocated from the cytoplasm into the nucleus 1 to 3 h after bacterial challenge (Fig 7C). We then extracted cytoplasmic and nuclear proteins from the intestine, and examined the subcellular distribution of STAT by western blotting using an anti-STAT antibody. The results showed that levels of total STAT in the intestinal tissue remained unchanged in the untreated, PBS-challenged or bacteria-challenged shrimp (Fig 7D). However, when STAT levels were analyzed separately in the cytoplasm and nucleus of the intestinal cells, when compared with the STAT levels in the cytoplasm of intestinal cells from PBS-challenged and untreated control shrimp, the STAT level at 3 h post-bacterial challenge was relatively lower (Fig 7E). Consistently, STAT was only detected in the nucleus of intestinal cells in the bacteria-challenged shrimp (Fig 7E). Taken together, these results suggest that challenge with bacteria and bacterial polysaccharides induce STAT phosphorylation and translocation into the nucleus at 3 h post-challenge in both hemocytes and intestinal cells, and indicate that bacterial challenge can activate the JAK/STAT signaling pathway in shrimp. To confirm that the JAK/STAT pathway is involved in the antibacterial response, we knocked down *STAT* by RNAi (Fig 7F), and comparatively analyzed bacterial clearance and the survival rate of the *STAT*-RNAi and *GFP*-RNAi control shrimp. Injection of *V*. *anguillarum* into the *STAT*-RNAi shrimp resulted in impaired bacterial clearance (Fig 7G), and their survival rate declined significantly compared with the *GFP*-RNAi control shrimp (Fig 7H). These results suggest that the JAK/STAT pathway plays an important role in antibacterial immunity in shrimp.

### The CCD of MjCC-CL mediates activation of the JAK/STAT signaling pathway

**Fig 8:**
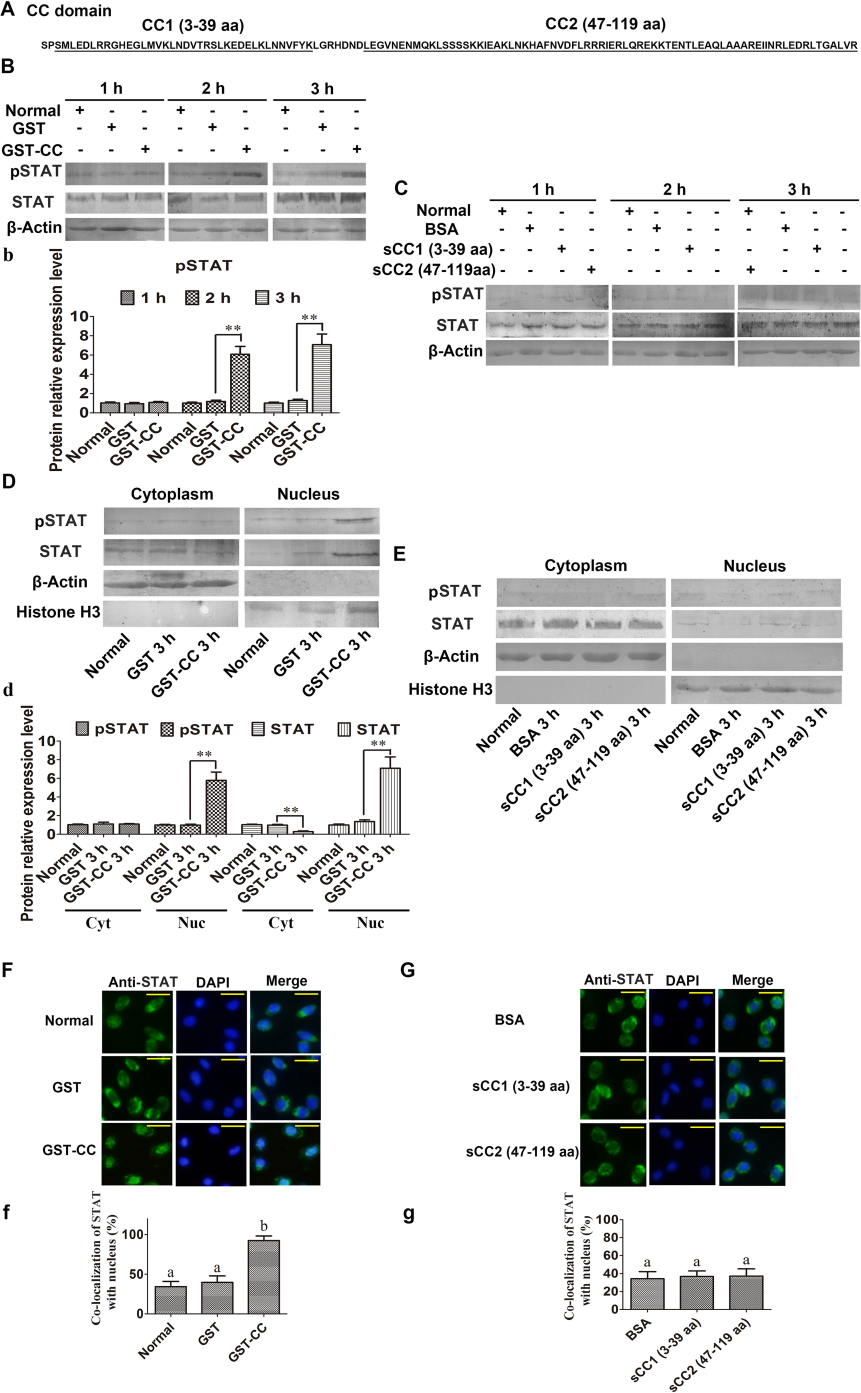
The CC domain of MjCC-CL activates the JAK/STAT pathway. (**A**) The sequence of CCD from MjCC-CL. (**B-C**) STAT phosphorylation in GST-CC-, sCC1 (3–39 aa)- and sCC2 (47–119 aa)-injected shrimp at 1, 2 and 3 h was determined by western blotting. (**b**) Statistic analysis of pSTAT with WCIF ImageJ software. (**D-E**) The subcellular distribution of STAT and STAT phosphorylation in GST-CC-, sCC (3–39 aa)- and sCC (47–119 aa)-injected shrimp at 3 h was detected by western blotting. BSA injection was used as the control. (**d**) Statistic analysis of pSTAT and STAT with WCIF ImageJ software. (**F-G**) STAT translocation in the hemocytes of GST-CC-, sCC (3–39 aa)- and sCC (47–119 aa)-injected shrimp at 3 h was detected using an immunocytochemical assay with a STAT-specific antibody. (**f-g**) Statistical analysis of F and G: the localization of STAT within the nucleus was analyzed using WCIF ImageJ software. Different letters indicate significant differences (*p* < 0.05) in one-way ANOVA analysis.

To further investigate the mechanism by which MjCC-CL mediates activation of the JAK/STAT pathway, we examined *in vivo* the potential role of the CCD on STAT phosphorylation and translocation into the nucleus of shrimp hemocytes. For this, the full length CCD (GST-CCD) expressed from *E*. *coli*, and two synthetic truncated forms, sCC1 (3–39 aa) and sCC2 (47–119 aa) (Fig 8A), were injected into shrimp and STAT phosporylation and translocation were analyzed as described above for the whole MjCC-CL. Injection of GST-CCD, resulted in significantly increased STAT phosphorylation at 2 and 3 h (Fig 8B-b), whereas no change in STAT phosphorylation levels at 1 h, 2 h, and 3 h after injection of sCC1 or sCC2 were detected (Fig 8C). Subsequently, cytoplasmic and nuclear proteins were extracted from hemocytes and the subcellular distributions of total STAT and pSTAT were assessed by WB using an anti-STAT and anti-pSTAT antibodies, respectively. The results showed that total STAT and pSTAT in the nucleus were increased at 3 h in hemocytes of the GST-CC-injected shrimp (Fig 8D-d), whereas no changes were observed in the sCC1- and sCC2 -injected shrimp (Fig 8E). We also assessed STAT translocation in hemocytes by immunocytochemical analysis, and the results showed that STAT translocated into the nucleus of the GST-CC-injected shrimp (Fig 8F and f), but no changes in sCC1- and sCC2-injected shrimp were observed (Fig 8G and g). The results strongly suggest that the CCD of MjCC-CL is responsible for activation of the JAK/STAT signaling pathway, and that the intact CCD structure is required for activity.

### MjCC-CL interacts with Domeless, the JAK/STAT pathway receptor on the hemocyte surface

**Fig 9:**
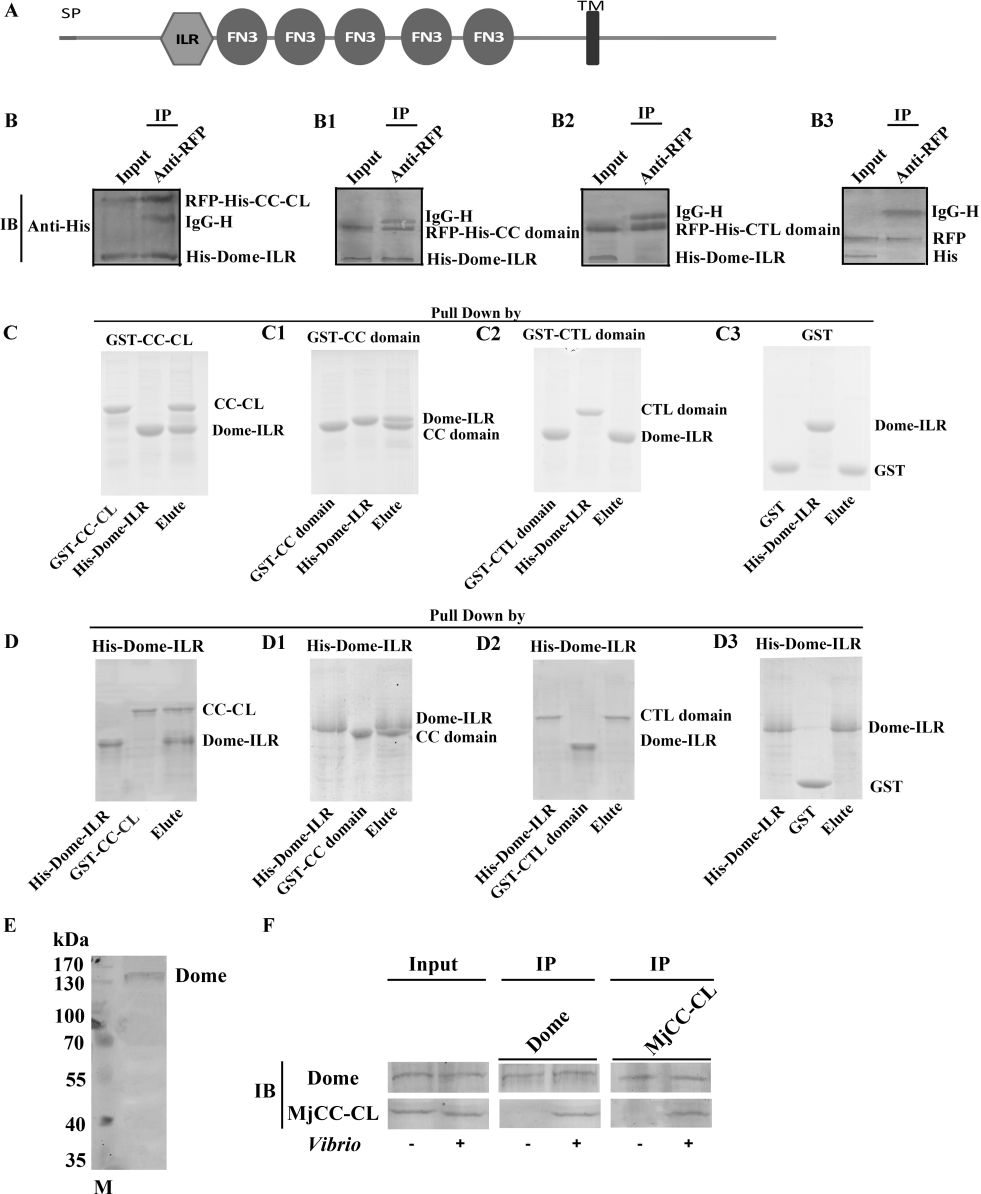
MjCC-CL interacts with the IL-R alpha domain of Dome. (**A**) The Dome receptor contains an SP, an IL-6R alpha domain (the receptor for interleukin 6), five FN3 domains (fibronectin type 3 domain) and a TM domain (transmembrane region). (**B**) MjCC-CL and the ILR domain of Dome were overexpressed in HaEpi cells, and co-IP was performed to detect the interaction between RFP-His-MjCC-CL and His-Dome-ILR using an anti-RFP antibody. The input lane showed that RFP-His-MjCC-CL and His-Dome-ILR were overexpressed in HaEpi cells before immunoprecipitation. Co-IP performed with the anti-RFP antibody showed the interaction between MjCC-CL and Dome-ILR. An anti-His antibody was used to detect the corresponding proteins. (**B1**) Co-IP was used to detect the interaction between the CCD of MjCC-CL and the ILR domain of Dome with an anti-RFP antibody. (**B2**) Co-IP was used to detect the interaction between the CTL domain of MjCC-CL and the ILR domain of Dome using an anti-RFP antibody. (**B3**) Control co-IP to detect the interaction of RFP with the His tag using anti-RFP antibody. (**C**) A GST pulldown assay was performed to detect the interaction between MjCC-CL and the ILR domain of Dome. (**C1-C2**) The interactions between the CCD of MjCC-CL (C1) or the CTL domain of MjCC-CL (C2) and the ILR domain of Dome were analyzed with a pulldown assay. (**C3**) GST pulldown was used as a control. (**D**) A His pulldown assay was performed to detect the interaction between the ILR domain of Dome and MjCC-CL. (**D1-D2**) The interactions between the ILR domain of Dome and the CCD of MjCC-CL (D1) or the CTL domain of MjCC-CL (D2) were analyzed using a pulldown assay. (**D3**) The interaction between the ILR domain of Dome and GST was used as a control. (**E**) Western blotting indicated the molecular masses of native Dome by detection with polyclonal antibody. The molecular weight markers were from Thermo Fisher Scientific, Lithuania. (**F**) Co-IP was performed using anti-Dome and anti-MjCC-CL antibodies in intestine after *Vibrio* challenge; MjCC-CL and Dome were detected by their corresponding antibodies. The experiments were repeated three times.

It has been established in *Drosophila* that Domeless (Dome) is the type I cytokine cell surface receptor involved in activation of the JAK/STAT pathway [19]. Thus, we investigated the possibility that in shrimp the Dome orthologue could also function as a lectin cell surface receptor, and therefore be involved in the MjCC-CL-mediated activation of the JAK/STAT pathway, upon an initial interaction of MjCC-CL with microbial pathogens. For this, we first cloned the kuruma shrimp Dome, (GenBank accession no. KX358405). Dome comprises a signal peptide, an interleukin 6 receptor (ILR) alpha domain, five fibronectin-type 3 (FN3) domains, and a transmembrane (TM) region (Fig 9A). We analyzed the potential interaction of MjCC-CL with Dome and cross-linking of bacteria by co-immunoprecipitation (co-IP), pulldowns, and bacterial binding assays. The pIEx-4-RFP plasmid with MjCC-CL or its CC and CTL domains was constructed and co-transfected into HaEpi cells [20] with the pIEx-4-RFP containing ILR domain of Dome, and a co-IP assay was performed to study the interaction between MjCC-CL and Dome. The results showed that MjCC-CL interacts with the ILR domain of Dome via its CCD (Fig 9B and 9B1) and that the CTL domain does not interact with ILR (Fig 9B2). There was no interaction between RFP and the His-tagged protein (Fig 9B3). Next, MjCC-CL and its CC and CTL domains, as well as the ILR domain of Dome, were expressed in *E*. *coli* (S1A–S1E Fig). A GST pulldown assay was performed to verify the interaction. The results showed that full-length MjCC-CL (Fig 9C) and the CCD of MjCC-CL (Fig 9C1) interacted with the ILR domain of Dome, but the CTL domain (Fig 9C2) and GST could not interact with ILR (Fig 9C3). The same results were obtained with His pulldown analysis (Fig 9D and 9D1-3). We then performed the Co-IP assay using antibodies specific for Dome or MjCC-CL (Fig 9E), and the results showed that upon *Vibrio* challenge Dome and MjCC-CL interacted with each other (Fig 9F). Taken together, the above results suggest that MjCC-CL functions both as a PRR of bacteria and a ligand of Dome, binding to bacteria with its CTL domain and cross-linking them to the Dome receptor with its CCD, thereby activating the of the JAK/STAT pathway.

### RNAi targeting Dome and JAK inhibits STAT phosphorylation and AMP expression

**Fig 10:**
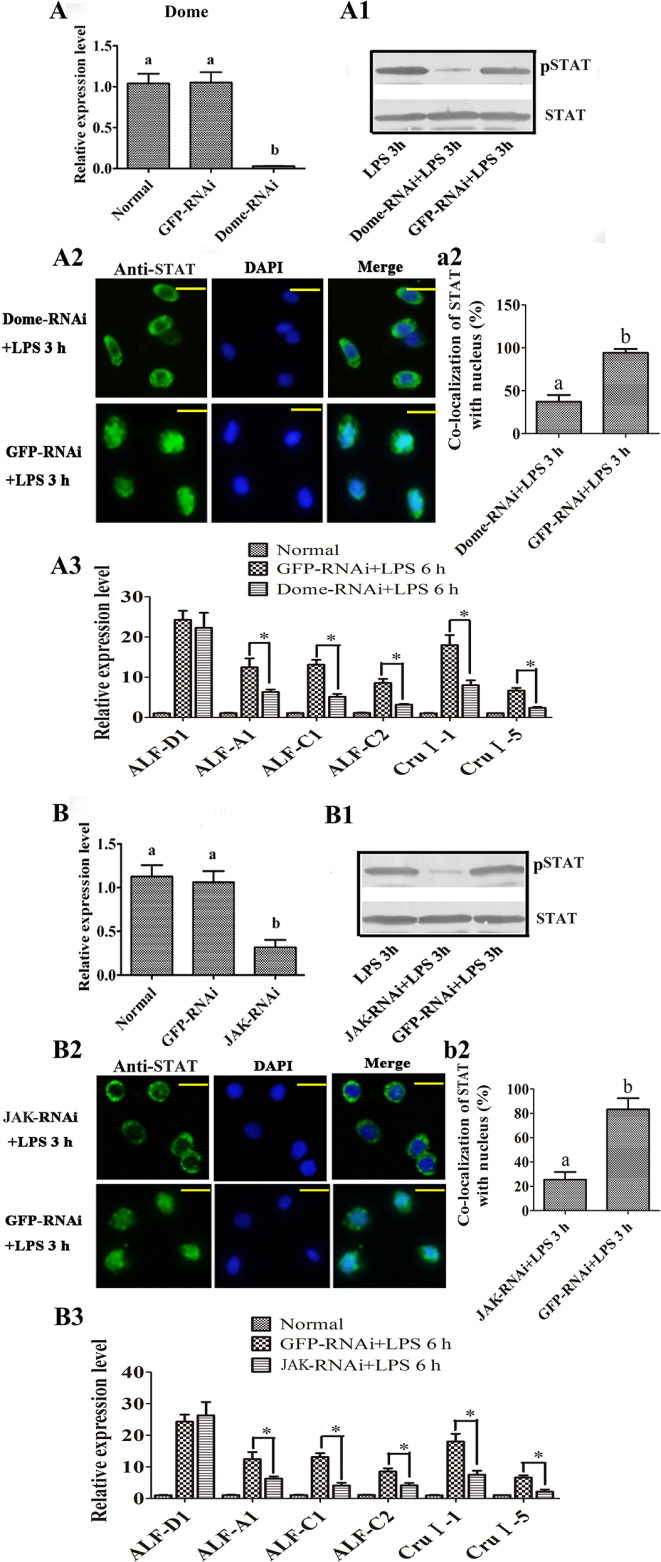
RNAi against *Dome* or *JAK* inhibited the phosphorylation and translocation of STAT and impaired the expression of five AMPs. (**A**) RNAi efficiency against *Dome* was analyzed by qRT-PCR. (**A1**) STAT phosphorylation in *Dome*-silenced shrimp was determined by western blotting. (**A2-a2**) STAT translocation in the hemocytes of *Dome*-silenced-shrimp was detected using an immunocytochemical assay with a STAT-specific antibody. a2 is the STATistical analysis of A2: the localization of STAT within the nucleus of hemocytes was analyzed using WCIF ImageJ software. (**A3**) The expression *of ALF-A1*, *ALF-C1*, *ALF-C2*, *ALF-D1*, *CruI-1* and *CruI-5* in *Dome*-silenced shrimp was analyzed by qRT-PCR. (**B**) The RNAi efficiency of *JAK* was analyzed with qRT-PCR. (**B1**) STAT phosphorylation in *JAK*-silenced shrimp was determined by western blotting. (**B2-b2**) STAT translocation in the hemocytes of *JAK*-silenced shrimp was determined using an immunocytochemical assay with a STAT-specific antibody. b2 is the Statistical analysis of B2: the localization of STAT within the nucleus was analyzed using WCIF ImageJ software. (**B3**) The expression of *ALF-A1*, *ALF-C1*, *ALF-C2*, *ALF-D1*, *CruI-1* and *CruI-5* in *JAK*-silenced shrimp was analyzed with qRT-PCR. Different letters indicate significant differences (*p* < 0.05) in a one-way ANOVA analysis. Differences in AMP expression were analyzed using a *t*-test (*p < 0.05).

JAK, the principal component of the JAK/STAT pathway, is present in the kuruma shrimp (GenBank accession no. KU213610). The architecture of JAK consists of a Band 4.1 homolog (B41) domain and an Src homology 2 (SH2) domain (S2A Fig). To investigate *in vivo* the potential role(s) of JAK and Dome in the shrimp immune response to bacterial challenge, we first examined the distribution and expression patterns of *JAK* and *Dome*. The results showed that *Dome* was mainly expressed in heart, gill and intestine, with lower expression in hemocytes, hepatopancrease and stomach. In contrast, *JAK* was similarly expressed in all tissues, with heart showing the lowest levels (S2B and S2C Fig). To confirm the role of the JAK/STAT pathway and specifically the functions of the key components *Dome* and *JAK*, we knocked them down by RNAi and analyzed STAT phosphorylation and AMP expression upon LPS challenge. Upon LPS challenge of the *Dome-*RNAi shrimp (Fig 10A), STAT phosphorylation decreased significantly in the intestine (Fig 10A1), STAT translocation into the nucleus was inhibited in the hemocytes (Fig 10A2 and a2), and the expression of *ALF-A1*, *ALF-C1*, *ALF-C2*, *CruΙ-1* and *CruΙ-5* was also significantly impaired (Fig 10A3). Similar results were obtained after treatment with RNAi targeting JAK (Fig 10B–10B3). The above results suggest that JAK and the MjCC-CL cell surface receptor Dome are key components of the JAK/STAT pathway and are involved in STAT phosphorylation and nuclear translocation, and AMP expression.

### MjCC-CL induces STAT3 phosphorylation in mouse macrophages

**Fig 11:**
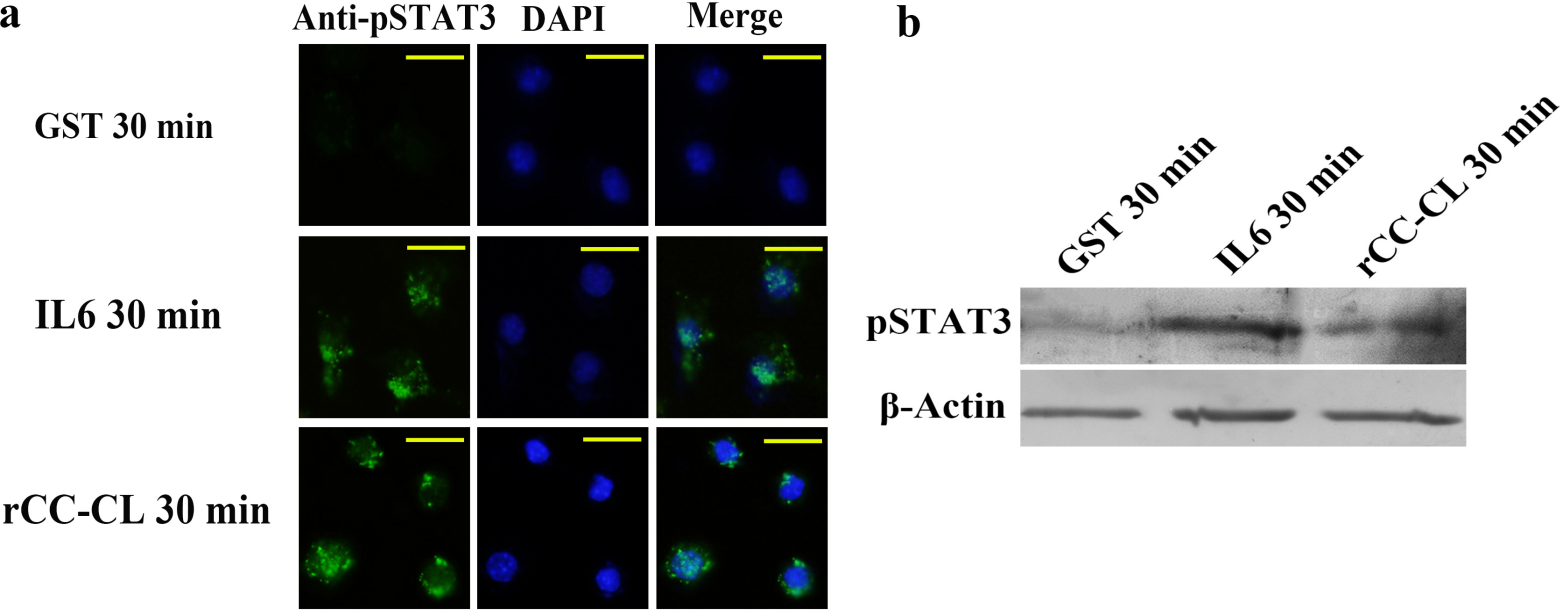
MjCC-CL induces STAT3 phosphorylation in mouse macrophages. (**A**). STAT3 phosphorylation in GST, IL6 or rMjCC-CL-treatment macrophages at 30 min was detected using an immunocytochemical assay with pSTAT3 antibody. (**B**). STAT3 phosphorylation in GST, IL6 or rMjCC-CL-treatment macrophages at 30 min was detected by western blotting. GST and IL6 were separately used as positive and negative control. Scale bars represented 20 μm.

To investigate the possibility that MjCC-CL could activate the JAK/STAT pathway in a heterologous system, we examined the MjCC-CL mediated JAK/STAT pathway activation in mouse macrophages, using STAT3 phosphorylation as an indicator, and IL6 as a positive control for pathway activation. After treatment with rMjCC-CL, the cells were collected and examined by immunocytochemical and WB analysis. The results showed like IL6, rMjCC-CL could induce STAT3 phosphorylation in mouse macrophages (Fig 11A and 11B). The results suggested that in a mammalian system MjCC-CL functions as a cytokine to activate the JAK/STAT pathway.

## Discussion

In this study we identified in the kuruma shrimp a novel chimeric CTL, MjCC-CL, which by a unique “self” protein-protein interaction at the cell surface plays a central role in the shrimp antibacterial immune response. The MjCC-CL protein comprises a typical CTLD that function as a PRR for “non-self” microbial glycans, and a CCD that by interacting with the Dome receptor at the hemocyte surface, directly activates the JAK/STAT signaling pathway to upregulate the expression of five AMPs.

Upon recognition of glycans on the surface of invading microbes via the canonical CTLD, soluble CTLs may participate in antibacterial responses in several ways, such as agglutinating and immobilizing the potential pathogens, functioning as opsonins to promote their phagocytosis, and by direct microbicidal activity or indirectly by activating enzymatic pathways leading to complement activation in vertebrates, or melanization by activation of the prophenoloxidase pathway in invertebrates [8]. We recently reported that the shrimp CTL MjHeCL directly regulates the AMP levels in plasma, which in turn maintain the homeostasis of the hemolymph microbiota [7]. MjHeCL only contains a CTLD, is structurally similar to CTLs from other invertebrate species, and is mainly expressed in hemocytes at relatively high levels that are not affected by experimental microbial challenge. In contrast, in addition to the CTLD, MjCC-CL displays a CCD rich in α-helix content with overall similarity to the mammalian IL10, and that a phylogenetic analysis finds clustering with vertebrate CTLs. Further, MjCC-CL was detected in all tested tissues and its expression was significantly upregulated by microbial infection. Although both MjHeCL and MjCC-CL regulated AMP expression, given the structural differences between the two CTLs, this likely to take place through different mechanisms, and aimed at very different functional outcomes: while the former maintains homeostasis of the internal microbiota, the latter is key to immune responses for exogenous infectious challenge. Most importantly, our findings in this study revealed a novel mechanism by which a CTL activates the JAK/STAT signaling pathway and regulates AMP expression.

The JAK/STAT pathway was originally identified as a cytokine signaling pathway in mammals [21], and its relevance in the regulation of both innate and adaptive immunity has been widely recognized [13]. The JAK/STAT pathway consists of three main components: cytokine receptors at the cell surface, Janus kinases (JAKs), and signal transducers and activators of transcription (STATs). In mammals, many cell surface cytokine receptors, four JAKs (JAK1, JAK2, JAK3 and TYK2) and seven STATs (STAT1, STAT2, STAT3, STAT4, STAT5A, STAT5B, and STAT6) have been identified [22]. Further, over fifty cytokines and growth factors, including interferons, interleukins and colony-stimulating factors, have been identified as indirect activators of the JAK/STAT pathway via cell surface receptors and mediate various immune responses to infection [23–25]. Although the JAK/STAT signaling pathway is ubiquitous in vertebrates, it can also be found as an intact pathway in some invertebrate taxa [26]. Thus, an evolutionarily conserved function of the JAK/STAT signaling pathway in immune responses in humans and insects, including fruit flies and mosquitos, has been suggested [15–18]. A complete JAK/STAT pathway is found in *Drosophila* and mosquito [12, 18, 27]. In mollusks [28] and echinoderms [29], only some components have been identified and it remains unclear whether they have a complete JAK/STAT pathway. Other organisms, such as *Caenorhabditis elegans* and *Dictyostelium*, do not have a completely functional JAK/STAT cassette, but homologs of some JAK/STAT pathway proteins have been found [30, 31]. However, there are no JAKs in *Dictyostelium*; instead, STAT activation is mediated by G protein-coupled receptors [32].

In *Drosophila*, Toll signaling and IMD pathways control the humoral immune response to bacterial or fungal infections, leading to the production of several AMPs [33–35], and the primary function of the JAK/STAT pathway is control of the cellular immune response [36]. The *Drosophila* JAK/STAT signaling pathway comprises two receptor-like molecules: one is Dome, which shows weak similarities to the cytokine-binding modules of the vertebrate IL6 receptor family and functions as the receptor of the pathway. Another is Latran (or Eye transformer), which has similarity to Dome and is encoded by a predicted gene, *CG14225*. Latran was reported to be a negative regulator of *Drosophila* JAK/STAT signaling [37, 38]. Cytokine-like proteins called Upds are ligands that bind to Dome and consequently activate the JAK/STAT pathway [19]. In contrast to the well-characterized Toll and IMD pathways in *Drosophila* immunity, however, relatively little is known about the transcriptional responses induced by the JAK/STAT pathway in humoral immune response.

In shrimp, the main components of the JAK/STAT pathway have been identified. Only one STAT, the key component of the pathway, is present in various shrimp species, including *Penaeus monodon* [39], *Fenneropenaeus chinensis* [40] and *Marsupenaeus japonicus* [41]. The shrimp STAT is similar to the mammalian STAT5. In mammals, STAT5 participates in the regulation of a wide range of physiological processes of the cell, including proliferation, differentiation, survival, apoptosis, and others [42, 43]. A single receptor domeless (Dome), which shares functional and sequence similarity with the mammalian cytokine class I receptors, has been identified in *Litopenaeus vannamei* [44]. A JAK has also been reported in *L*. *vannamei* [45]. Unlike the antiviral function of the JAK/STAT pathway in mammals and insects, white spot syndrome virus (WSSV) uses a shrimp STAT as a transcription factor to enhance viral gene expression in host cells [46], and the pathway is helpful and beneficial for WSSV replication [44, 47]. However, another report has shown that silencing of the shrimp JAK causes higher mortality and increased viral load in *L*. *vannamei*, and the pathway has antiviral function [45]. In contrast to the function of JAK/STAT pathway in antiviral responses, relatively little is known about the function of the pathway in antibacterial defense.

In our study, the ligand of the JAK/STAT pathway receptor, MjCC-CL, was identified and found to contain a CCD with sequence similarity to IL10 and a CTLD. Through protein secondary structure prediction and SWISS-MODEL analysis, the CCD was also predicted to have a highly α-helical nature, which is highly similar in overall structure with cytokine, IL10 in mammals. The fundamental property of coiled coils is their stability. The biological functions resulting from coiled-coil stability are related with membrane fusion, transmembrane signal transduction, and interaction with different proteins [48]. The CCD binds to the Dome receptor to activate the JAK/STAT pathway. The CTL domain of the MjCC-CL binds both Gram-positive bacteria and Gram-negative bacteria by binding to different polysaccharides, including LPS and PGN, on the bacterial surface. Numerous C-type lectins have been identified in shrimp and other invertebrates, and these lectins have functional diversity [8, 49]. Therefore, this study identifies an example of direct activation of the JAK/STAT pathway and provides novel ways to identify new ligands of the JAK/STAT pathway in invertebrates. MjCC-CL from shrimp also induces STAT3 phosphorylation in mouse macrophages, suggesting that MjCC-CL function as a cytokine in mammals. Although the principal components of the JAK/STAT pathway have been identified in other invertebrate species, the ligands capable of activating the pathway have been described only in *Drosophila*. Three Upds that activate the JAK/STAT pathway in flies were identified, including Upd and Upd3 [15, 50]. Although the Upds bear no sequence similarity to cytokines, the predicted highly α-helical nature is consistent with an overall structure that could be similar to cytokines. In our study, the lectin MjCC-CL not only activates the JAK/STAT pathway in shrimp hemocytes, but can also function as a cytokine by activating JAK/STAT pathway in mammalian macrophages.

In the present study, significant changes in the principal components of the shrimp JAK/STAT signaling pathway were observed after challenge with bacteria or their surface glycans, such as LPS and PGN. These challenges induced the phosphorylation and translocation of STAT in shrimp, and up regulation of expression of five AMPs via JAK/STAT signaling. Further, our study revealed a putative STAT-binding site in the promoter sequence of an AMP, CruI-1. The JAK/STAT pathway is associated with various immune responses, including anti-viral and anti-bacterial responses [12, 51]. The first evidence that the JAK/STAT pathway is involved in invertebrate immunity came from studies performed in *Anopheles* mosquitoes, which indicated that the STAT protein accumulates in the nucleus after immune challenge [52]. A subsequent study found that the JAK/STAT pathway participates in hematopoiesis and the cellular immune response in *Drosophila* [12]. Gene expression profile studies have identified a subset of *Drosophila* immune-responsive genes that are regulated by the JAK/STAT pathway, namely the genes encoding the complement-like protein Tep2 and the Turandot stress genes, but the exact function of these genes in immunity remains elusive [53]. In shrimp, STAT is upregulated in shrimp challenged with bacteria, WSSV, poly IC and PGN [54]. STAT can enhance the replication of WSSV in *Penaeus monodon* [46].

**Fig 12:**
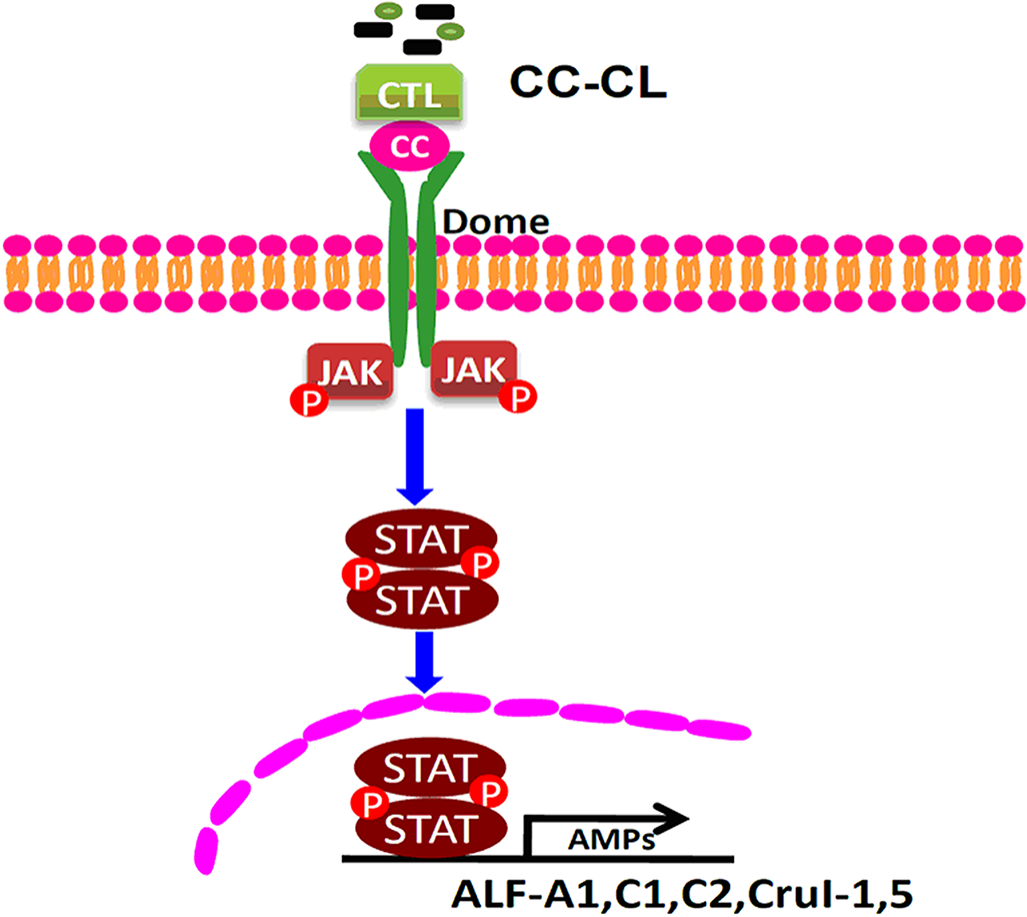
MjCC-CL directly activates the JAK/STAT signaling pathway to regulate AMP expression. The CTL domain of MjCC-CL recognizes different bacteria, and the CCD of MjCC-CL interacts with the ILR domain of the Dome receptor. The transcription factor STAT is phosphorylated and translocates into the nucleus, inducing the AMP expression. JAK/STAT pathway activation is responsible for the clearance of invading bacterial pathogens by regulating AMP expression.

In conclusion, the components of the shrimp JAK/STAT pathway were identified, including a ligand, MjCC-CL; a receptor, Dome; a kinase, JAK; and a transcription factor, STAT, and the mechanistic aspects of their interactions upon immune challenge were elucidated, as schematically illustrated in Fig 12. Upon bacterial challenge (including Gram-positive or Gram-negative bacteria), MjCC-CL can recognize the pathogens’ surface glycans, *via* the CTLD. MjCC-CL then interacts *via* the CCD with the receptor Dome to activate the JAK/STAT pathway and induce the phosphorylation and translocation of STAT, which in turn regulates the expression of *ALF-C1*, *ALF-C2*, *ALF-D1*, *CruΙ-1* and *CruΙ-5*. Taken together, our results suggest that MjCC-CL functions both as a PRR for recognition of infectious pathogens, and as the ligand of Dome to activate JAK/STAT signaling, and that the MjCC-CL/Dome/JAK/STAT pathway plays an important role in the anti-bacterial response by regulating AMP expression in shrimp. Thus, our study of the shrimp MjCC-CL revealed a striking functional difference with vertebrates, in which the JAK/STAT pathway is indirectly activated by cell death and stress signals through cytokines or growth factors. Instead, by cross-linking microbial pathogens with the cell surface receptor Domeless, a lectin directly activates the JAK/STAT pathway, which plays a central role in the shrimp antibacterial immune responses by upregulating expression of selected AMPs.

## Materials and methods

### Cloning, sequencing, homology modeling, and phylogenetic analysis of MjCC-CL

The full-length cDNA sequence of MjCC-CL was obtained from shrimp intestine by transcriptomic sequencing. The sequence of MjCC-CL was amplified by RT-PCR using corresponding primers (S2 Table) and re-sequenced for confirmation. Similarity analysis was conducted using BLASTx (http://www.ncbi.nlm.nih.gov/). The corresponding cDNA was conceptually translated, and the deduced proteins were predicted using ExPASy (http://www.expasy.org/). Domain architecture prediction of the proteins was performed using SMART (http://smart.embl-heidelberg.de/). Homology modeling of MjCC-CL was performed using SWISS-MODEL (https://swissmodel.expasy.org/). MEGA 5 was used for phylogenetic analysis.

### Infectious challenge of shrimp, collection of tissues, and preparation of RNA

Healthy kuruma shrimp (*M*. *japonicus*; about 10 g each) were obtained from a fish market in Jinan, Shandong Province, China, and were acclimated in a laboratory aquarium tanks with aerated seawater at 23°C for 3 days before the infectious challenge. For this, each shrimp was injected in the abdomen with either 20 μl of bacteria (*Vibrio anguillarum*, 1 × 10^7^ CFU in PBS, 1 ml) or PBS alone. For hemocyte collection, hemolymph was extracted from three challenged or control shrimp with a syringe containing 1 ml cold anticoagulant buffer at 4°C (0.45 M NaCl, 10 mM KCl, 10 mM EDTA and 10 mM HEPES, pH 7.45) and immediately centrifuged at 800 g for 10 min (4°C). Organs (heart, hepatopancreas, gills, stomach and intestine, were collected, and total RNA was extracted with TRIzol reagent (Cwbio, Beijing, China).

### Cloning of the main components of JAK/STAT pathway

The full-length cDNA sequences of Dome, Jak and Stat were obtained from shrimp intestine by transcriptomic sequencing. All sequences were amplified by RT-PCR using specific primers (S2 Table) and the amplicons sequenced and analyzed by BLASTx (http://www.ncbi.nlm.nih.gov/) for sequence confirmation. Prediction of the domain architecture of the corresponding proteins was performed using SMART (http://smart.embl-heidelberg.de/).

### Tissue distribution and expression profiles of MjCC-CL, Dome, JAK and STAT

The mRNA tissue distribution of five genes was analyzed using semi-quantitative RT-PCR with the primers RT-F and RT-R for *MjCC-CL*, *Dome*, *JAK and STAT* (S2 Table). RNA from the hemocytes, heart, hepatopancreas, gills, stomach, and intestine was used in this assay. *β-actin* was used as the control with the primers ActinF and ActinR (S2 Table). qRT-PCR was used to detect the expression profiles of five genes after *V*. *anguillarum* infection following a previously described method [7]. The qRT-PCR was programmed at 95°C for 10 min, followed by 40 cycles at 95°C for 10 s and 60°C for 60 s. The plate was read at 78°C for each cycle. The expression profiles of *MjCC-CL*, *Dome*, *JAK* and *STAT* were detected in the hemocytes and intestine challenged with *V*. *anguillarum*. All experiments were repeated at least three times using individual templates. The obtained data were evaluated using the 2^-ΔΔCt^ method, as described previously [55], and statistically analyzed; significant differences in the unpaired sample *t*-test were accepted at *p*< 0.05.

### RNA interference

The 3’-terminal sequence (about 500 bp) for siRNA of MjCC-CL, Dome and STAT were amplified by the primers Fi and Ri linked to the T7 promoter (S2 Table) and were used as templates for the synthesis of *dsRNA*. The cDNA fragment of GFP used for *dsGFP* synthesis was amplified using the primers GFP-Fi and GFP-Ri (S2 Table). The *dsRNA* was synthesized using T7 polymerase (Fermentas, USA) based on the method of Wang et al [56]. The RNA interference (RNAi) assay was performed as described in previous reports [57]. dsRNA (20 μg) for *MjCC-CL*, *Dome* or *STAT* was injected into the abdominal segment of each shrimp. To enhance the RNAi effect, a second injection was performed 12 h after the first injection. *dsGFP* was used as a control. The intestine was collected from the shrimp 24 h after the second injection, and total RNA was extracted and assessed by qRT-PCR using the primers RT-F and RT-R (S2 Table) to evaluate the efficacy of the RNAi. To screen potential AMPs regulated by JAK/STAT signaling, AMP expression in challenged shrimp after receiving *STAT*-RNAi plus challenge were analyzed. The RNA interference was followed previous mentioned method. The shrimp were divided into four groups: one control group received PBS injection and the other RNAi groups were injected with *V*. *anguillarum*, lipopolysaccharides (LPS; *E*. *coli*, Sigma), peptidoglycan (PGN, *S*. *aureus*, Sigma; PGN, *B*. *subtilis*, Sigma). AMPs, including ALF-A1, ALF-C1, ALF-C2, ALF-D1 [58], CruΙ-1 and CruΙ-5, were detected by qRT-PCR with specific primers (S2 Table) after 6 h of bacterial challenge. The above AMPs were detected after *STAT*-knockdown and then challenged with LPS. *dsGFP* was used as the control.

### Bacterial clearance assay

After the RNAi method was established, a bacterial clearance assay was performed. Shrimp were separated into three groups and injected with *dsMjCC-CL*, *dsSTAT* or *dsGFP* as a control. Then, the three groups were injected with *V*. *anguillarum* (20 μl, 1 × 10^9^ CFU). Thirty minutes after bacteria injection, shrimp hemolymph was collected, diluted and then cultured on solid LB plates overnight. The numbers of bacterial colonies were counted. The assay was repeated three times.

### Shrimp survival assay

Shrimp (30 shrimp per group, about 10 g each) were divided into three groups to evaluate the shrimp survival rate after *STAT* and *MjCC-CL* knockdown and *V*. *anguillarum* infection. *dsGFP* was used as a control. After *STAT* and *MjCC-CL* were knocked down by dsRNA injection, all shrimp were injected with *V*. *anguillarum* (20 μl, 1 × 10^10^ CFU in PBS, 1 ml). The number of dead shrimp was monitored every day, and the survival rates of the three groups of shrimp were calculated. The experiments were repeated three times. The data were statistically analyzed by *t*-test, and a difference was considered to be significant at *p*< 0.05.

### Recombinant expression, purification and antibody preparation of MjCC-CL

MjCC-CL, the CCD and CTLD of MjCC-CL, CTL2 (as a control C-type lectin), and the ILR domains of Dome and STAT were recombinantly expressed in *Escherichia coli*. The sequences of the above five proteins were amplified from shrimp hemocytes using the primers ExF and ExR (S2 Table). The PCR procedure was as follows: one cycle at 95°C for 3 min; 35 cycles at 94°C for 30 s, 55°C for 45 s, and 72°C for 45 s; and one cycle at 72°C for 10 min. The PCR products were then cloned into the pET32a (Novagen) or pGEX4T-1 (GE Healthcare) vectors. The recombinant proteins were purified by affinity chromatography using His-Bind resin (Ni2+-resin; Novagen, Darmstadt, Germany) or GST-resin (GenScript, Nanjing, China) following the manufacturer’s instructions. MjCC-CL, STAT antiserum preparation was performed as previously described [59]. The truncated forms of CCD (3–39 aa) and (47–119 aa) from MjCC-CL (sequences were shown in Fig 6A) were synthesized by DgPeptidesCo., Ltd (Hangzhou, China), and named as synthesized truncated CC1, sCC1 (3–39 aa) and synthesized truncated CC2, sCC2 (47–119 aa). Dorsal and Relish expression and antiserum preparation were carried out as previously described [60, 61].

### Recombinant protein binding assay

Gram-positive bacteria (*B*. *subtilis* and *S*. *aureus*) and Gram-negative bacteria (*E*. *coli* and *V*. *anguillarum*) were used to test the binding activity of recombinant MjCC-CL and the CTL and CCDs of MjCC-CL. Bacteria were cultured in 2–4 mL of Luria-Bertani (LB) medium (1% tryptone, 0.5% yeast extract, and 1% NaCl) overnight and then gathered by centrifugation at 6000 *g* for 5 min. After washing three times with TBS, the bacteria were resuspended in TBS and adjusted to an OD600 of 1.0. The bacteria (400 μL) in TBS were incubated with purified rMjCC-CL, rCTLD and rCCD (100 μg) for 60 min at 28°C with rotation, collected by centrifugation, and then washed four times with TBS. Finally, the bound proteins were eluted with 7% SDS for 1 min and subjected to 12.5% SDS-PAGE. The proteins in the gel were transferred to a nitrocellulose membrane for western blot analysis. An anti-histidine antibody (ZSGB Bio, Beijing, China, 1:3000 dilution in TBS containing 5% nonfat milk) was used as the primary antibody, and secondary antibody was alkaline phosphatase-conjugated horse anti-mouse IgG (ZSGB Bio, Beijing, China, 1:10,000 dilution in TBS containing 5% nonfat milk). Native MjCC-CL protein was purified from shrimp intestine according to a previous report [7] and also incubated with four bacteria. The mixture was gently rotated at 28°C for 1 h. Bacterial pellets were collected by centrifugation at 6000 *g* for 5 min, washed three times with Tris buffer (pH 8.0) and then analyzed by western blotting.

An enzyme-linked immunosorbent assay (ELISA) was used to test the direct binding activity of rMjCC-CL, rCTLD and rCCD to different bacterial cell wall components. LPS from *E*. *coli* and PGN from *S*. *aureus* and *B*. *subtilis* separately were chosen for the assay. Each well of the microplate was coated with 2 μg of the polysaccharide and incubated at 37°C overnight. The microplate was incubated at 60°C for 30 min, blocked with BSA (1 mg/mL, 200 μL) at 37°C for 2 h, and washed with TBS (200 μL). Purified rMjCC-CL, rCTLD and rCCD (final concentration 0–20 μg/mL in TBS with 0.1 mg/mL BSA) was added to each well of the coated plates and incubated at room temperature for 3 h. The plate was then washed four times with TBS, and alkaline phosphatase-conjugated horse anti-mouse IgG (1:3000 dilution in binding buffer containing 0.1 mg/mL BSA) was added (100 μL per well) and incubated at 37°C for 2 h. After the plate was washed four times with TBS, the color was developed with p-nitro-phenyl phosphate (1 mg/mL in 10 mM diethanolamine and 0.5 mM MgCl_2_) at room temperature for 30 min. The OD value was read at 405 nm. Each binding assay was performed three times. The dissociation constants (Kd) and maximum binding (Bmax) parameters were calculated by GraphPad Prism version 5.00 software for Windows (San Diego, CA, USA).

### Antibacterial activity assays

Gram-positive bacteria (*B*. *subtilis* and *S*. *aureus*) and Gram-negative bacteria (*E*. *coli* and *V*. *anguillarum*) were used to test the potential antibacterial activity of recombinant MjCC-CL. Each protein (10, 30, 50, 100 μg) was added to a 96-well culture plate which contained 180 μL of mid-log phase bacteria (2 × 10^5^ CFU) cultured in Poor Broth (1% tryptone, 0.5% NaCl (w/v) and pH 7.5). The plate was incubated for 24 h at 28°C and the absorbance at 600 nm was measured using an ELX800 Universal Microplate Reader (Bio-Tek Instruments, INC) to evaluate the bacterial concentration. The assays were repeated thrice. GST protein was used as negative control.

### Overexpression assay and translocation detection of transcription factors

Recombinant MjCC-CL was used for the “overexpression” assay. rMj-CC-CTL (20 μg per shrimp) was injected into shrimp for 1 and 2 h. GST was used as control. Hemocytes were then collected for immunocytochemical assay and intestine protein was extracted for western blotting to detect Dorsal, Relish and STAT translocation into nucleus. rMj-CC-CTL, sCC1 (3–39 aa) and sCC2 (47–119 aa) were also injected into shrimp to detect STAT phosphorylation and translocation into nucleus by western blotting assay and immunocytochemical assay. To confirm the function of MjCC-CL in JAK/STAT pathway activation, rMj-CC-CTL and rCTL2 incubated with LPS (4 μg) were injected into shrimp for 3 h. GST was used as control. Hemocytes were then collected for immunocytochemical assay and intestine protein was extracted for western blotting to detect Dorsal, Relish and STAT translocation into nucleus. The AMPs were also assessed in intestine in rMj-CC-CTL- and rCTL2-injection shrimp at 6 h.

### Western blot analysis

Western blotting was used to detect STAT phosphorylation with the commercial anti-STAT5 (phospho Tyr694) antibody (Abcam, USA). Tissue proteins were obtained from the hemocytes and intestine of normal shrimp and bacterially challenged shrimp. Cytoplasmic proteins and nuclear proteins were extracted using a Nuclear Protein Extraction Reagent Kit (BioTeke, China) following the manufacturer’s instructions. The samples were separated by 12.5% SDS-polyacrylamide gel electrophoresis following the Laemmli method [62]. The proteins in the gel were then transferred onto nitrocellulose membranes. The membranes were blocked for 1 h with 3% non-fat milk in TBS (10 mM Tris-HCl, pH 7.5, 150 mM NaCl) and incubated with 1/100 diluted antiserum against STAT, phosphorylated STAT, Dorsal, Relish or β-actin in TBS with 3% non-fat milk for 2 h. Then, alkaline phosphatase-conjugated goat anti-rabbit IgG (1/10,000 diluted in TBS) was added after washing to remove the free, nonspecifically binding antiserum, and the membranes were incubated for 2 h. The membrane was dipped in the reaction system (10 ml of TBS with 45 μl of NBT and 35 μl of BCIP) in the dark for 5 min to visualize the signal.

### Immunocytochemical analysis

Hemolymph obtained from shrimp was fixed with 1 ml of a mixture containing anticoagulant (pH 7.4) and 4% paraformaldehyde and then centrifuged at 600 *g* for 10 min at 4°C. The collected hemocytes were deposited onto a glass slide, washed with PBS (140 mM NaCl, 10 mM sodium phosphate, pH 7.4) and incubated in 0.2% Triton X-100 at 37°C for 5 min. After washing with PBS, the hemocytes on the glass slides were blocked with 3% BSA (30 min, 37°C) and incubated with anti-pSTAT, anti-STAT, anti-Dorsal or Anti-Relish (1:400 in 3% BSA) overnight at 4°C. The hemocytes were then washed with PBS and incubated with 3% BSA for 10 min; the Alexa Fluor 488-conjugated second antibody to rabbit (1:1,000 ratio, diluted in 3% BSA) was then added, and the samples were incubated for 1 h at 37°C in the dark. After being washed three times, the hemocytes were incubated with 4’-6-diamidino-2-phenylindole dihydrochloride (DAPI, AnaSpec Inc., San Jose, CA; 1 μg/ml in PBS) for 10 min at room temperature and washed six times. Fluorescence was observed under an Olympus BX51 fluorescence microscope (Shinjuku-ku, Tokyo, Japan). WCIF ImageJ software was used to analyze the colocalization of STAT and DAPI-stained nuclei in hemocytes.

### Inhibitor injection

As STAT in *M*. *japonicus* is clustered together with STAT5 in human (S1 Fig), STAT5 inhibitor, (573108-10MG, Merck) (2 μg) was used to inject into each shrimp and then challenged by LPS. DMSO injection was used as a control. The intestine was collected for protein and RNA extraction at 3 and 6 h after LPS challenge. The phosphorylation level of STAT was analyzed by western blotting with anti-pSTAT antibody, and the AMP expression was detected by qRT-PCR at 6 h after LPS challenge.

### Genome walking and chromatin immunoprecipitation (ChIP)

The promoter sequences of antimicrobial peptides regulated by the JAK/STAT pathway were cloned using Genome Walker Kits (Takara, Japan) following the manufacturer’s instructions. The specific primers against CruΙ-1 (G-CruΙ-1-1, G-CruΙ-1-2 and G-CruΙ-1-3) used for genome walking are listed in S2 Table. ChIP was performed following previously described methods [63, 64] using the primers CruΙ-1 RTF/R for CruΙ-1 (S2 Table).

### Electrophoretic mobility shift assay (EMSA)

The shrimp intestine was lysed, and STAT protein was purified using anti-STAT antibody- CNBr-activated Sepharose 4B (60 mg; Amersham Biosciences AB, Uppsala, Sweden). The digoxigenin-labeled probes (sense, 5'-GCGTAAGGTTTTCTTGGAATA-3'; antisense, 5'-TATTCCAAGAAAACCTTACGC-3') were synthetized and labeled by Sangon Company (China). Two micrograms of purified proteins was mixed with 3 μl of 5× binding buffer (Beyotime Institute of Biotechnology, Shanghai, China) for 10 min and incubated with 20 fmol of digoxigenin-labeled probe for 20 min. In competition experiments, unlabeled probe was pre-incubated with the relevant proteins for 10 min before the Dig-labeled probe was added and incubated for 20 min at room temperature. The reaction solution was run on a 6% polyacrylamide/0.5× TBE gel at 80 V, and the samples were transferred onto a nylon membrane (IMMOBILON-NY+, Millipore, Milford, MA, USA). The membrane was first blocked with blocking buffer for 30 min and then incubated with anti-Dig phosphatase antibody (1:10000 in blocking solution; Roche, Germany) for 1h. The signal was visualized with 5-bromo-4-chloro-3-indolyl phosphate and nitroblue tetrazolium chloride.

### Overexpression of MjCC-CL and the ILR domain of Dome in HaEpi cells and co-immunoprecipitation

To analyze the interaction of MjCC-CL with Dome, co-immunoprecipitation was performed after these molecules were overexpressed in HaEpi cells. The appropriate cDNA sequences encoding whole MjCC-CL or the CC region and CTL domain of MjCC-CL were amplified with primers (S2 Table) and inserted into the pIEx-4-RFP plasmid (with a C-terminal red fluorescent protein tag); then, the ILR domain of Dome was also amplified and inserted into the pIEx-4 plasmid (with a His tag). HaEpi cells [20] were incubated in a 6-well tissue culture plate containing 2 ml of Grace’s medium with 10% FBS at a density of 70% to 90%. Before transfection, the cells were pre-incubated in Grace’s medium for 1 h. Afterward, 8 μg of vector DNA and 8 μg of DNAfectin transfection reagent (Tiangen, Beijing, China) were mixed, suspended in 200 μl of Grace’s medium and incubated for 20 min; then, this solution was added to the medium in the culture plate. After 12 h, the cells were re-fed in Grace’s medium containing 10% FBS and cultured for an additional 48 h. The cells were then harvested and washed twice with ice-cold 1× PBS. Afterward, the cells were re-suspended in SDS-lysis buffer (1% SDS, 10 mM EDTA, 50 Mm Tris-HCl, pH 8.1), and the lysates were pre-cleared with protein A resin at 4°C for 1 h and incubated with anti-GFP antibody at 4°C overnight. The mixture was then incubated with protein A resin at 4°C. After 2 h, the complex was washed three times and analyzed by western blotting with anti-His antibody.

### Pull-down assay and co-immunoprecipitation (Co-IP) assay

To further confirm the interaction of MjCC-CL with Dome, whole MjCC-CL, the CC and CTL domains of MjCC-CL with GST tags and the ILR domain of Dome with a His tag were expressed in *E*. *coli*, and GST-pulldown and His-pulldown were performed. Recombinant proteins (30 μg) were added to 20 μl of glutathione resin (for GST-tagged proteins) or charged Ni-NTA beads (for His-tagged proteins) and incubated at room temperature for 2 h with slight rotation. The mixture (resin and binding proteins) was washed three times by centrifugation at 500 *g* for 3 min to remove the unbound proteins. The test protein with a His tag or GST tag was added into the mixture and was gently rotated at room temperature for 2 h. After the resin was washed three times, bound proteins were eluted and analyzed by SDS-PAGE.

Co-immunoprecipitation (Co-IP) assay. Proteins from shrimp intestine were extracted with lysis buffer (150 mM NaCl, 1.0% Nonident-P40, 0.1% SDS, 50 mM Tris [pH 8.0]) and incubated with protein A for 10–15 min to remove non-specific binding proteins. Then, proteins were incubated with antibodies specific for Dome or MjCC-CL for 3 h at room temperature, after which the mixture was incubated with protein A for 3 h at room temperature, and the pellet washed with PBS five times. The resulting pellet (bound protein, antibody and protein A) was analyzed by western blot.

### Cell culture, cell treatment and pSTAT3 detection

The mouse primary peritoneal macrophages were obtained from Dr. Cheng-Jiang Gao laboratory in Medical School of Shandong University, the procedure of cell isolation following previous report [65]. The cells were cultured at 37°C under 5% CO2 in DMEM supplemented with 10% FCS (Invitrogen Life Technologies), 100 U/ml penicillin, and 100 μg/ml streptomycin. IL6 (ProSpec, Israel), rGST and rMjCC-CL (20 ng/ml) were added in the cell culture for 30 min. Then the cells were collected and used for immunocytochemical assay and western blotting. PSTAT3 antibody (Abcam, USA) was used as the first antibody to detect the STAT3 phosphorylation in mouse macrophages.

## Supporting information

S1 Fig Recombinant proteins of GST, GST-MjCC-CL, GST-IL domain, GST-CTL domain and His-Dome-ILR were expressed and purified from *E. coli*. (**A**) GST expression and purification. (**B-E**) GST-MjCC-CL (**B**). GST-CC domain of MjCC-CL (**C**). GST-CTL domain of MjCC-CL (**D**). His-Dome-ILR (**E**). Lane M, protein marker; lane 1, proteins of *E*. *coli* with recombinant vectors before induction with IPTG; lane 2, protein marker; lane 1, proteins of *E*. *coli* with recombinant vectors after induction with IPTG; lane 3,purified protein.
(TIF)

S2 Fig The Dome, JAK and STAT domain architectures, and tissue distribution and expression patterns of *Dome* and *JAK* in shrimp. (**A**) JAK from *M*. *japonicus* contains a B41 (Band 4.1 homologues), a SH2 (Src homology 2 domain) and two protein kinase activity domains. STAT from *M*. *japonicus* contains a NTD (N-terminal domain), a CC (coiled-coil domain), a DB (DNA-binding domain), a LD (linker domain), a SH2 (SH2 domain) and a TAD (transactivation domain). (**B, C**) The tissue distribution of *Dome* (**B**) and *JAK* (**C**) were analyzed by qRT-PCR. *β-actin* was used as the control. The mRNA was extracted from the hemocytes, heart, hepatopancreas, gills, stomach, and intestines and used for reverse transcription and qRT-PCR analysis. (**B1, B2, C1, C2**) qRT-PCR was used to detect the time course of *Dome* (**B1, B2**) and *JAK* (**C1, C2**), expression in the hemocytes and intestines after challenge with *V*. *anguillarum*. Differences among the groups were analyzed using one-way ANOVA followed by Tukey’s multiple comparison *t*-test. Different letters indicate significant differences (*p* < 0.05).
(TIF)

S3 Fig Nucleotide sequence of CruI-1 promotor. Genome walking was used for obtaining genomic sequence of *CruΙ-1*. The binding sites of *CruΙ-1* genomic sequence were marked. NF-κB (Rel) binding site marked with gray; NF-κB (RelA) binding site marked with blue; Stat1/2 binding site marked with green; Stat5a/5b binding site marked with red; a TATA box marked with yellow; a transcriptional start site marked with purple.
(TIF)

S1 Table Bacteria maximum binding parameter (*B*max) and dissociation constant (*K*d) of CC-CTL to different polysaccharides. Nonlinear regression analysis showed that the binding of rMjCC-CL, rMjCC and rMjCTL to different polysaccharides fitted a two-site binding model (*R*^2^ > 0.96). *B*max of *Mj*CC-CTL binding to LPS from *E*. *coli* and PGN from *S*. *aureus* and PGN from *B*. *subtilis* were 0.948, 1.047, and 0.9301, respectively.
(TIF)

S2 Table Sequences of the primers used in this study. (TIF)
