# Supplementary figures and images for: Binding of a C-type lectin’s coiled-coil domain to the Domeless receptor directly activates the JAK/STAT pathway in the shrimp immune response to bacterial infection
Source: PLoS Pathog. 2017 Sep 20;13(9):e1006626. doi: 10.1371/journal.ppat.1006626 (PMC5645147; doi:10.1371/journal.ppat.1006626)

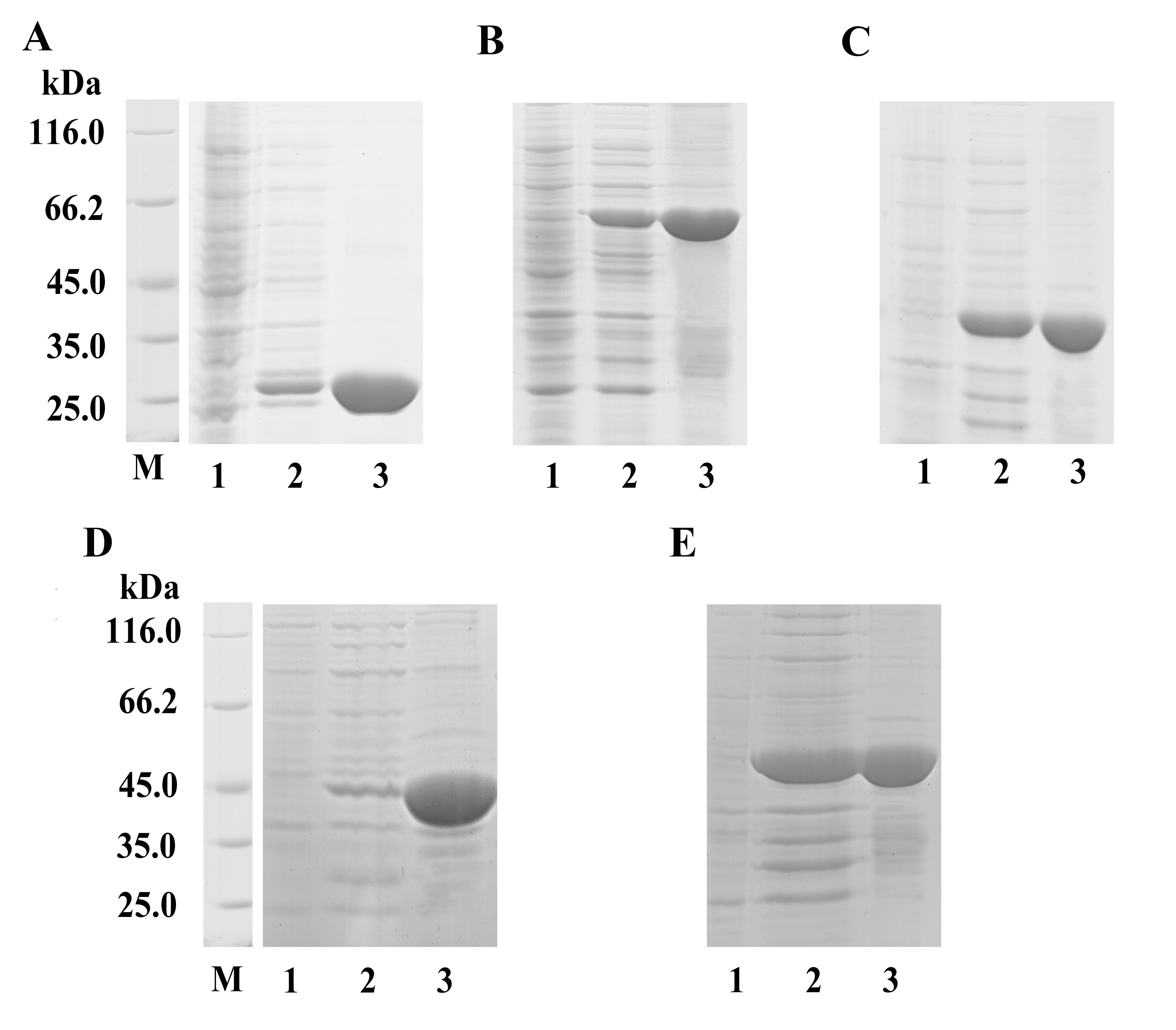

Supplement: S1 Fig — (A) GST expression and purification. (B-E) GST-MjCC-CL (B). GST-CC domain of MjCC-CL (C). GST-CTL domain of MjCC-CL (D). His-Dome-ILR (E). Lane M, protein marker; lane 1, proteins of E. coli with recombinant vectors before induction with IPTG; lane 2, protein marker; lane 1, proteins of E. coli with recombinant vectors after induction with IPTG; lane 3,purified protein. (TIF) [file ppat.1006626.s001.tif]

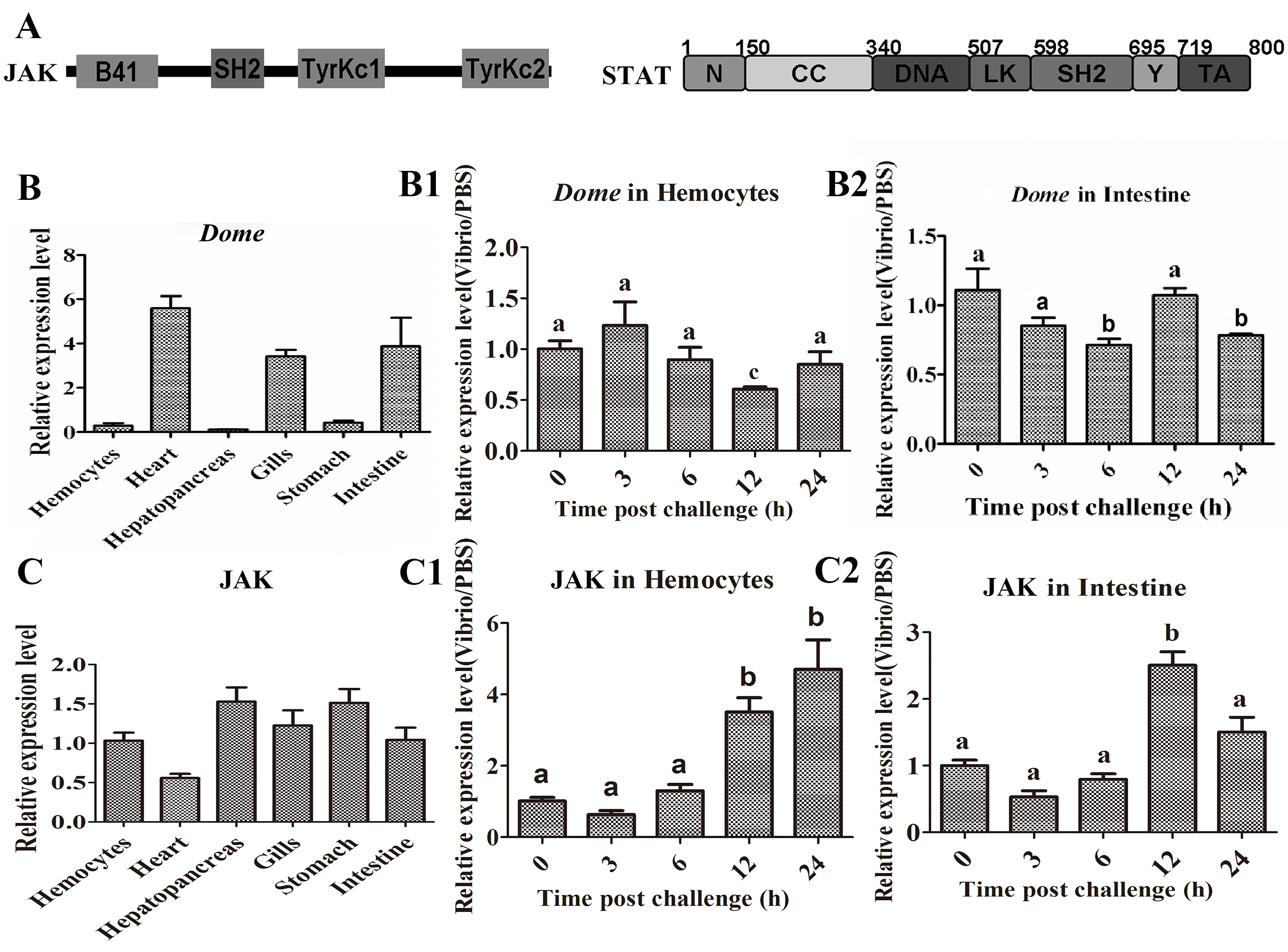

Supplement: S2 Fig — (A) JAK from M. japonicus contains a B41 (Band 4.1 homologues), a SH2 (Src homology 2 domain) and two protein kinase activity domains. STAT from M. japonicus contains a NTD (N-terminal domain), a CC (coiled-coil domain), a DB (DNA-binding domain), a LD (linker domain), a SH2 (SH2 domain) and a TAD (transactivation domain). (B, C) The tissue distribution of Dome (B) and JAK (C) were analyzed by qRT-PCR. β-actin was used as the control. The mRNA was extracted from the hemocytes, heart, hepatopancreas, gills, stomach, and intestines and used for reverse transcription and qRT-PCR analysis. (B1, B2, C1, C2) qRT-PCR was used to detect the time course of Dome (B1, B2) and JAK (C1, C2), expression in the hemocytes and intestines after challenge with V. anguillarum. Differences among the groups were analyzed using one-way ANOVA followed by Tukey’s multiple comparison t-test. Different letters indicate significant differences (p < 0.05). (TIF) [file ppat.1006626.s002.tif]

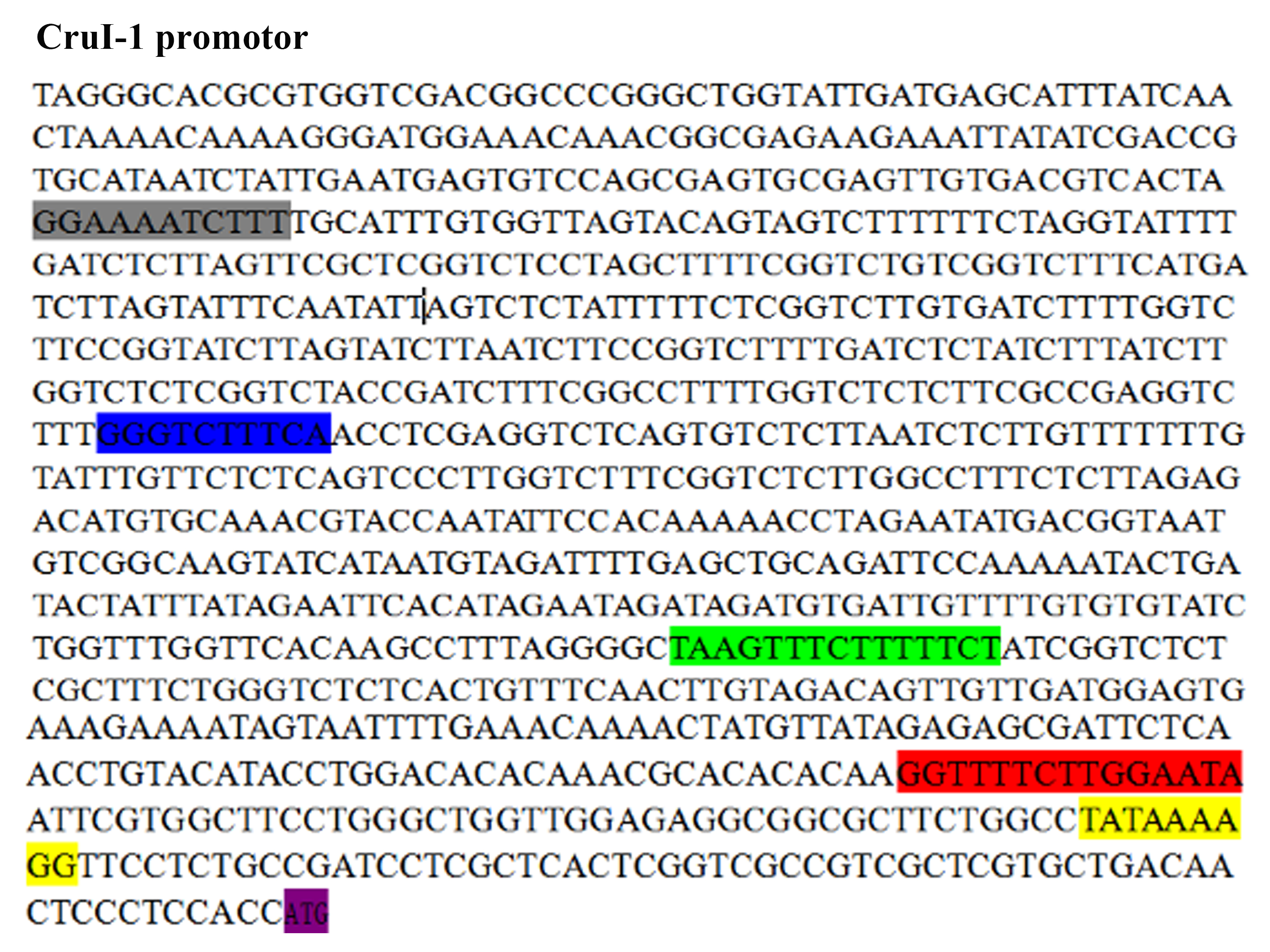

Supplement: S3 Fig — Genome walking was used for obtaining genomic sequence of CruΙ-1. The binding sites of CruΙ-1 genomic sequence were marked. NF-κB (Rel) binding site marked with gray; NF-κB (RelA) binding site marked with blue; Stat1/2 binding site marked with green; Stat5a/5b binding site marked with red; a TATA box marked with yellow; a transcriptional start site marked with purple. (TIF) [file ppat.1006626.s003.tif]

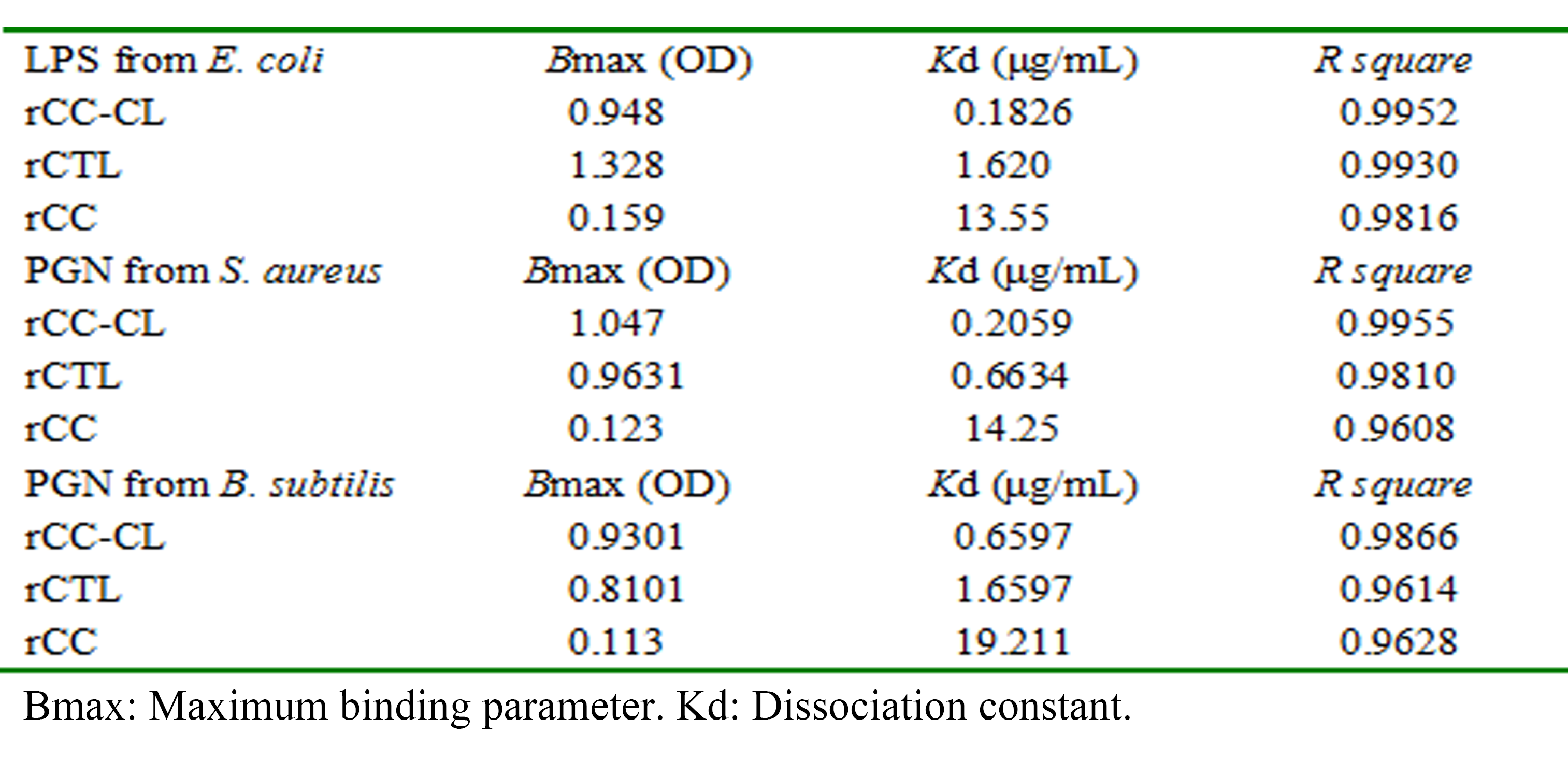

Supplement: S1 Table — Nonlinear regression analysis showed that the binding of rMjCC-CL, rMjCC and rMjCTL to different polysaccharides fitted a two-site binding model (R2 > 0.96). Bmax of MjCC-CTL binding to LPS from E. coli and PGN from S. aureus and PGN from B. subtilis were 0.948, 1.047, and 0.9301, respectively. (TIF) [file ppat.1006626.s004.tif]

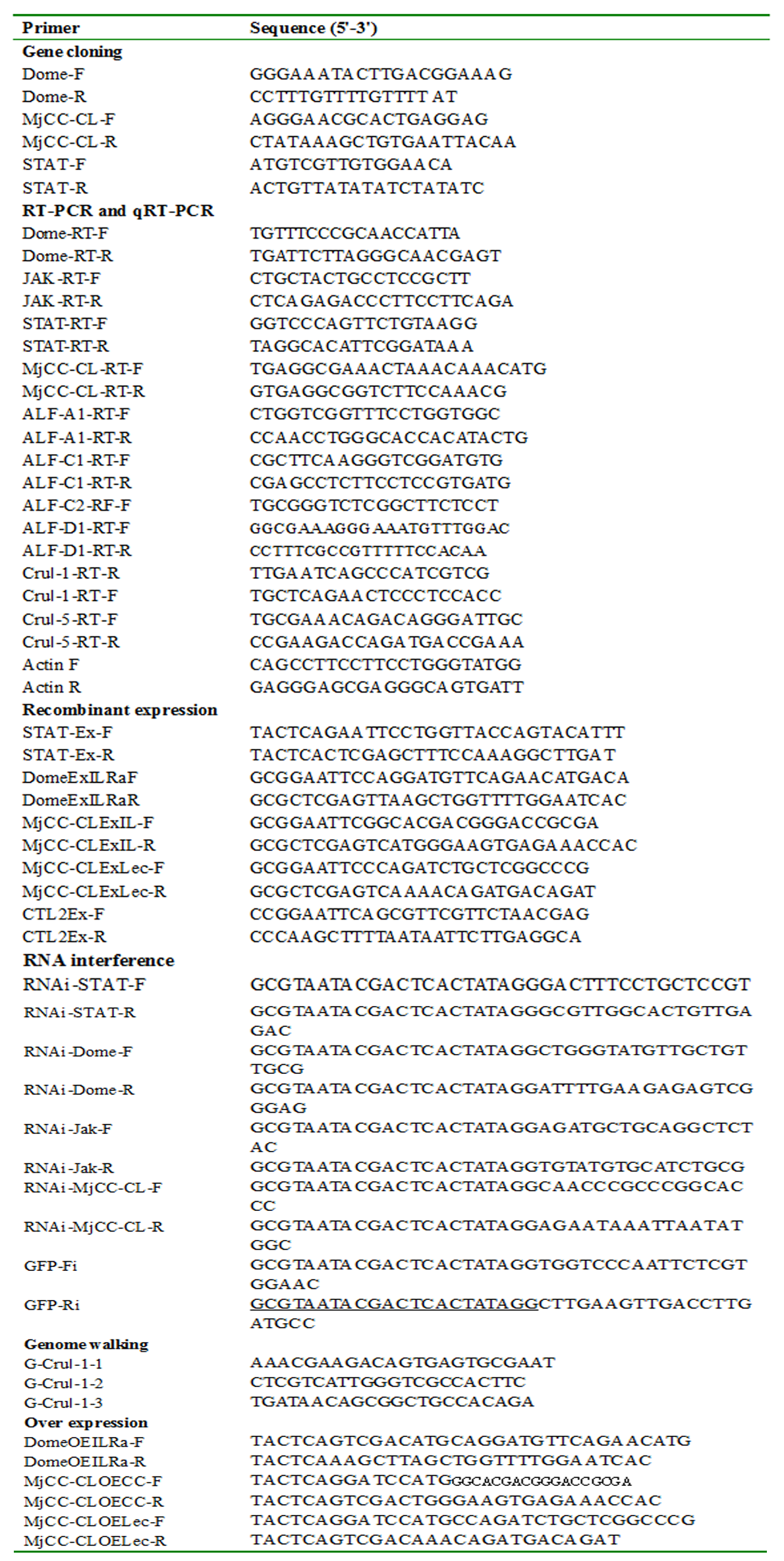

Supplement: S2 Table — (TIF) [file ppat.1006626.s005.tif]
